# Porous Hydrogen-Bonded Organic Frameworks

**DOI:** 10.3390/molecules22020266

**Published:** 2017-02-13

**Authors:** Yi-Fei Han, Ying-Xue Yuan, Hong-Bo Wang

**Affiliations:** 1 Key Laboratory of Optoelectronic Chemical Materials and Devices of Ministry of Education, School of Chemical and Environmental Engineering, Jianghan University, Wuhan 430056, Hubei, China; 15200828166@163.com (Y.-F.H.); 15200821478@163.com (Y.-X.Y.); 2 College of Chemistry and Chemical Engineering, Central South University, Changsha 410083, Hunan, China

**Keywords:** hydrogen bonds, organic porous frameworks, supramolecular self-assembly

## Abstract

Ordered porous solid-state architectures constructed via non-covalent supramolecular self-assembly have attracted increasing interest due to their unique advantages and potential applications. Porous metal-coordination organic frameworks (MOFs) are generated by the assembly of metal coordination centers and organic linkers. Compared to MOFs, porous hydrogen-bonded organic frameworks (HOFs) are readily purified and recovered via simple recrystallization. However, due to lacking of sufficiently ability to orientate self-aggregation of building motifs in predictable manners, rational design and preparation of porous HOFs are still challenging. Herein, we summarize recent developments about porous HOFs and attempt to gain deeper insights into the design strategies of basic building motifs.

## 1. Introduction

Highly ordered porous solid-state architectures formed by supramolecular self-assembly via non-covalent interactions (such as metal coordination or hydrogen bonds, etc.) have attracted increasing interest owing to their broad applications in areas such as sensing, biomedical imaging, drug delivery, heterogeneous catalysis, proton conduction, and gas separation and storage [1,2,3,4,5,6,7,8,9,10,11,12,13,14,15]. Although porous hydrogen-bonded organic frameworks (HOFs) have been proposed for about two decades, it is difficult to stabilize such frameworks with permanent porosities once the solvent guests are removed. As HOFs can be easily purified and recovered by recrystallization, the design and preparation of HOFs with outstanding porosity are still challenging.

**Figure 1:**
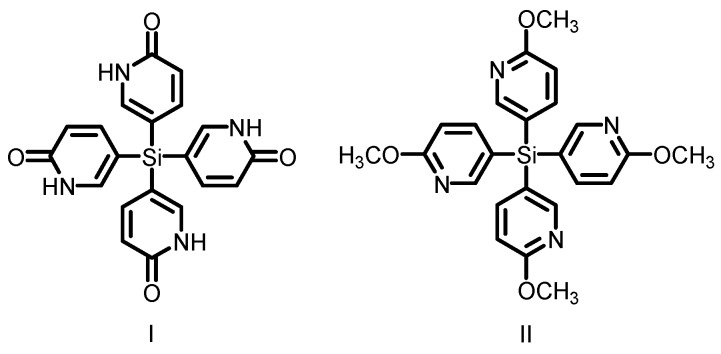
Structures of the building motifs **I** (left) and **II** (right).

In the beginning, “molecular tectonics” was defined as a process for the “chemical construction of higher-order architectures” [16]. Recently, molecular tectonics has been considered as a powerful strategy for the construction of porous crystalline covalent organic frameworks. According to this strategy, the basic building blocks of porous HOFs are special molecules with binding sites involving non-covalent directional interactions. More importantly, these directional interactions play indispensible roles in the formation of open networks with significant porosity. For example, the crystallization of tetrakis(1,2-dihydro-2-oxo-5-pyridyl)silane (**I**) (see Figure 1) is directed by the characteristic hydrogen bonding of multiple pyridone groups, affording open networks with approximately 60% of the volume available for the inclusion of guests. In contrast, the structure of compound **II** (see Figure 1) is close-packed, devoid of guests, and held together primarily by combination of van der Waals and aromatic interactions [17].

Generally speaking, HOF building motifs mainly consist of two indispensible parts, namely a scaffold (or backbone) and hydrogen bonding interaction sites (or sticky sides). In some elegant examples, there also exist linkers (or spacers) between the rigid backbones and sticky sides. Rigid scaffolds, which constitute the basic framework of HOF materials, usually exhibit highly rigid structural features, thereby inhibiting the inevitable free rotation or vibration of building motifs. Then all hydrogen bonding sticky sides are attached to rigid backbones via rational designed synthesis routes. From our perspective, the structural symmetries of rigid cores and synthetic convenience of motifs play crucial roles in predetermination of the number of hydrogen bonding sites in binding motif. Finally, all binding sites in these motifs assemble with adjacent moieties to accomplish the fabrication of HOFs via hydrogen bonding interactions.

Considering that HOFs are connected together by non-covalent hydrogen bonds, herein we speculate that hydrogen bonding strength between binding sites may play crucial roles in affecting porosity of HOF materials. Therefore, we intend to summarize recent progress in HOFs in this review. Depending on categories of hydrogen bonding sites, HOFs are deliberately classified into various groups for the purpose of discussing conveniently. Additionally, we do not illustrate examples in this review by complicated principles or explanations of crystallography. On the contrary, we attempt to elucidate HOF examples from perspectives of hydrogen bonding assembly. Due to the limited space, some classical and elegant examples are not included in this review.

## 2. The Main Content of This Review

According to the diversity of hydrogen bonding sticky sides, we elegantly divide the content of this review into eight categories. In the following context, we will discuss properties of diverse HOF materials.

### 2.1. Hydroxyl or Amine Groups as Hydrogen-Bonded Motifs for HOFs

**Figure 2:**
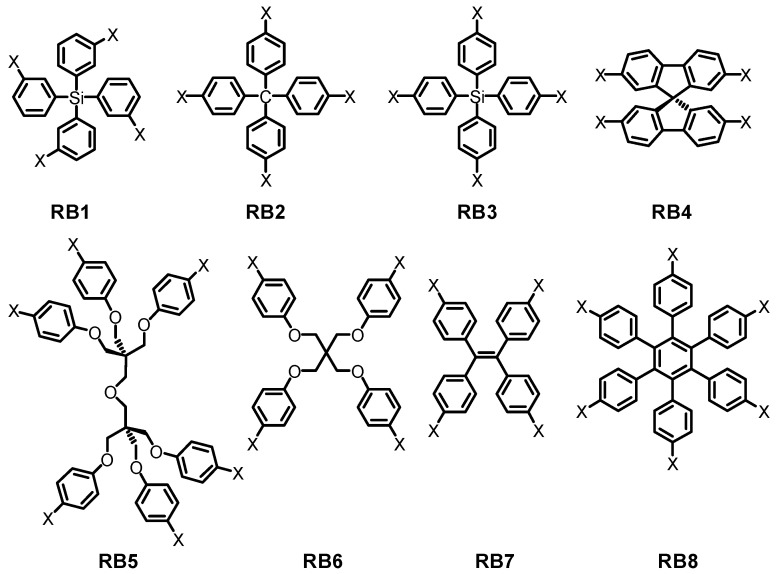
Structures of rigid and flexible scaffolds **RB1**–**8**.

In earlier studies, Wuest found that assembly of counterparts tetrakis(3-hydroxylphenyl)silane and tetrakis(4-hydroxylphenyl)silane immediately resulted in the formation of diamondoid networks [18]. However, neither interpenetration or sufficient volume for guests is observed in HOFs assembled from tetrakis(3-hydroxylphenyl)silane, as evidenced by crystalline architectures. In contrast, tetrakis(4-hydroxylphenyl)methane exhibits zigzag channels with 28% porosity [18]. Later on, Wuest and other prominent supramolecular chemists devoted themselves to another backbones, namely **RB1**–**8** (see Table 1 and Figure 2). They initially investigated diverse NH_2_ tetrasubstituted backbones (**RB4**–**8**) [19,20,21,22]. Unfortunately, X-ray diffraction examinations of crystals demonstrated that, although HOFs possessing **RB4** and **RB7** backbones were less packed, the porosities of the corresponding HOFs (see Table 1) were generally below 18%. In exceptional cases, **HOFs** bearing **RB4** and **RB8** were confirmed to be particularly suitable for inhibiting close packing of crystals. For example, the overall space volumes available for guest encapsulation in both HOFs (see Figure 2, X = OH) were calculated to be approximately 43% and 48%, respectively.

Apart from OH sticky sides, other self-complexation groups such as NH_2_, COOH, CONH_2_, or CN groups are also deliberately integrated into **RB8**, leading to the formation of open hydrogen-bonded networks with a wide range of porosities. As compared to other scaffolds listed in Figure 2, the unique advantages of **RB4** and **RB8** are partially attributed to intrinsic highly rigidity of backbones, thereby inhibiting unfavorable free mobility or severely interpenetration of building motifs during crystal growth.

**Figure 3:**
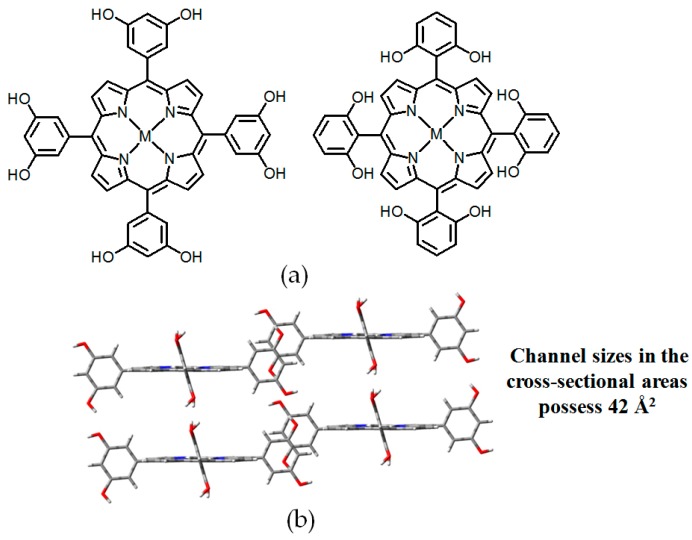
(**a**) Structures of building motifs of **H1a**–**1f** (M = 2H, Zn(II), or Mn(III)); (**b**) Top view of the crystalline packing structure of **H1a** (CH_3_COOC_2_H_5_ are omitted for clarity).

**Figure 4:**
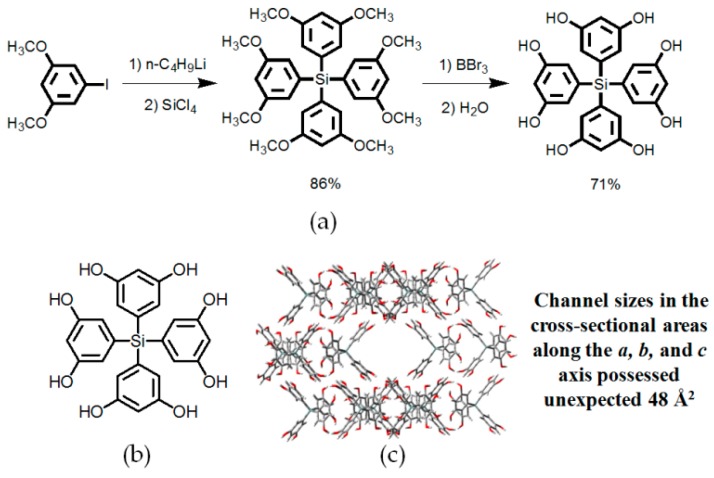
(**a**) Synthetic strategy of the building motif **2**; (**b**) Structure of the building motif **2**; (**c**) Crystalline packing structure of the porous network of **H2**.

In term of strengthening association affinities of sticky sides, many elegant examples have been reported in the previous literature. For example, Suslick and coworkers designed and synthesized a collection of crystalline hydrogen-bonded supramolecular networks based on porphyrin scaffolds (see Figure 3a) [26]. In their design, four resorcinol moieties were deliberately incorporated into the *meso*-positions of 5,10,15,20-tetrakis(3,5-dihydroxylphenyl)porphyrin to ensure that the porphyrin cores assemble together via multiple hydrogen bonds. Notably, XRD characterization of single crystals demonstrated that the resulting topology exhibits an amazing 63% porosity when benzonitrile guests are encapsulated (see Figure 3b). In crystalline **H1a**, four resorcinol moieties are nearly vertical with the central porphyrin plane by O–H⋯O hydrogen bonds with adjacent resorcinol units in *meso* positions, thereby leading to the formation of interconnected cavities without unfavorable π-π stacking. With exceptionally large size of 42 Å in cross-sections of channels, the volume for guests was estimated above 60%. To the best of our knowledge, functionalized porphyrin compounds are difficult to synthesize due to their tedious preparation and great difficulties in purification. Aoyama reported another example of a resorcinol unit-based hydrogen bonding network with 62% porosity, which was further applied in stereoselective alkyne catalysis or Diels-Alder reactions [1,27]. Inspired by the great advantages of resorcinol, Wuest and his-coworkers reported a novel motif, tetrakis(3,5-dihydroxylphenyl)silane, prepared in 61% overall yield via two facile steps (see Figure 4a) [28]. As validated by XRD studies, the porosities of **H2** (see Figure 4b) were determined to be 45% (hexane/ethyl acrylate) and 52% (hexane/THF), respectively. In sharp contrast, tetrakis(3,5-dimethoxyphenyl)silane grown from dichloromethane (DCM) assembles in close-packed manners, as the precursor is indeed incapable of generating open hydrogen-bonded networks owing to a lack of efficient orientation groups. Accordingly, there are two factors which may explain the poor porosity performance of these HOFs. It is likely that weak hydrogen bonding between monosubstituted phenyl groups may be incapable of resisting evident contraction of opened networks once all guests are removed. In addition, reliable rigid cores should have the ability to prevent themselves from close-packed in crystalline architectures. Indeed, these basic principles have been confirmed to be really efficient in later discoveries.

**Figure 5:**
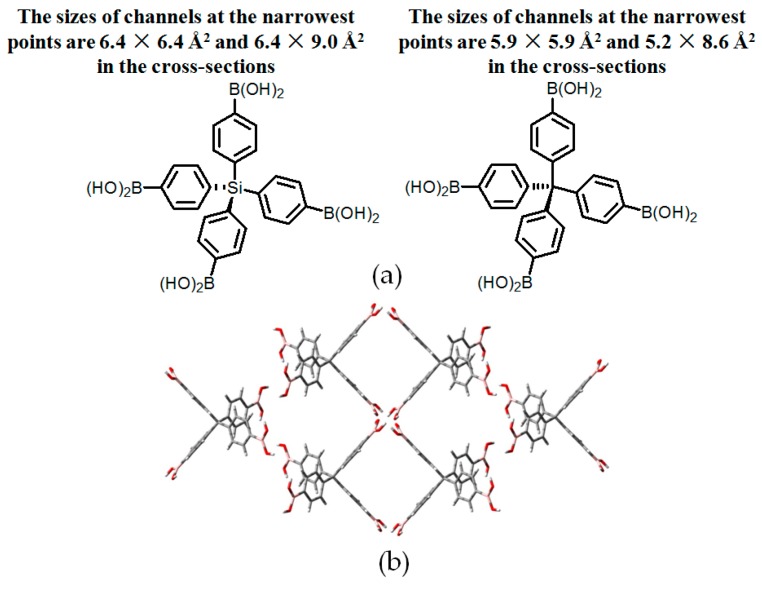
(**a**) Structures of building motif **3** (left) and **4** (right); (**b**) Top view of crystal network of **H4**.

**Figure 6:**
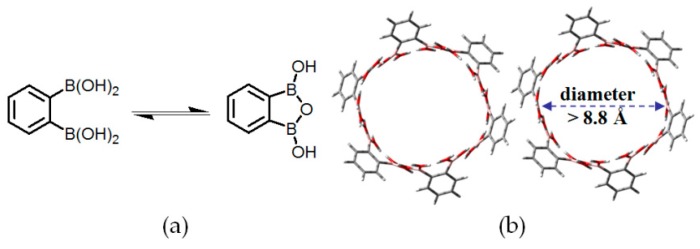
(**a**) Reaction equilibrium between 1,2-diphenylene-diboronic acid and **1-sanh** in water; (**b**) Hexagonal channels formed in the nanotubular structure of **H5**.

It is well known that derivatives of aromatic boronic acids possessing B(OH)_2_ groups are frequently utilized to facilitate occurrence of Suzuki coupling reactions with decent yield. Analogous to carboxylic acids, B(OH)_2_ is quite good at generating cyclic dimers in solution or the solid state. By integrating B(OH)_2_ groups into **RB2** and **RB3** (see Figure 5a), Wuest and his-coworkers found that **H3** and **H4** retained interconnected channels (see Figure 5b) and porosities of both diamond-like supramolecular networks were determined to be 60% and 64%, respectively, which significantly exceeded the aforementioned HOF geometries [29]. It is noteworthy that intercalated guests in channels can be efficiently exchanged without losing crystalline properties. Additionally, B(OH)_2_-containing building motifs are allowed to assemble into more sophisticated geometries. For example, 1,2-diphenylenediboronic acid has a strong propensity to convert into semianhydride (**1-sanh**) via intramolecular dehydration between adjacent B(OH)_2_ groups (see Figure 6a). XRD analysis of crystalline **H5** revealed that every six cyclic semianhydride moieties were hold together via H–O⋯H hydrogen bonds to form well-organized macrocyclic rings. Notably, OH groups located at the rim of rings evidently facilitate fabrication of highly ordered nanotubes via self-complexation of neighboring macrocyclic rings (see Figure 6b) [30]. Interestingly, the diameter of channels in nanotube geometries were estimated above 8.8 Å even in the narrowest position and these channels were filled with water guests. On the basis of PLATON computational simulation, the precise average molar ratio of **1-sanh** and guest was calculated to be 1:1, which was in line with characterization results of thermo gravimetric analysis (TGA). Moreover, this porous network was further assembled together and stabilized by weak C–H⋯π interactions and B–O bonds.

### 2.2. Carboxylic Groups or Pyrazole Moieties as Hydrogen-Bonded Motifs for HOFs

The key in constructing HOFs is to figure out how HOFs self-assemble together in predictable and controllable manners. Analogous to B(OH)_2_, COOH is also able to form specific dimers via strong hydrogen bonding interactions [31]. After investigation of related HOF articles, we found that it was an ideal opportunity for us to elucidate how symmetric of rigid cores affect assemble of HOFs in controllable manners. For example, the tetrahedral motif adamantane-1,3,5,7-tetracarboxylic acid was designed and its assembly properties were carefully examined [32]. With four carboxylic acid groups in a tetrahedral vertex, this compound aggregates into an ordered crystalline structure to afford three-dimensional diamondoid network. Analogously, when tetrahedral scaffold is substituted with planar 1,3,5-trisubstituted benzene, the resulting *C*_3_-symmetric 1,3,5-benzenetricarboxylic acid motif eventually aggregates into hexagonal crystalline network, which was confirmed by reliable crystal geometries reported by Duchamp and Marsh [33].

**Figure 7:**
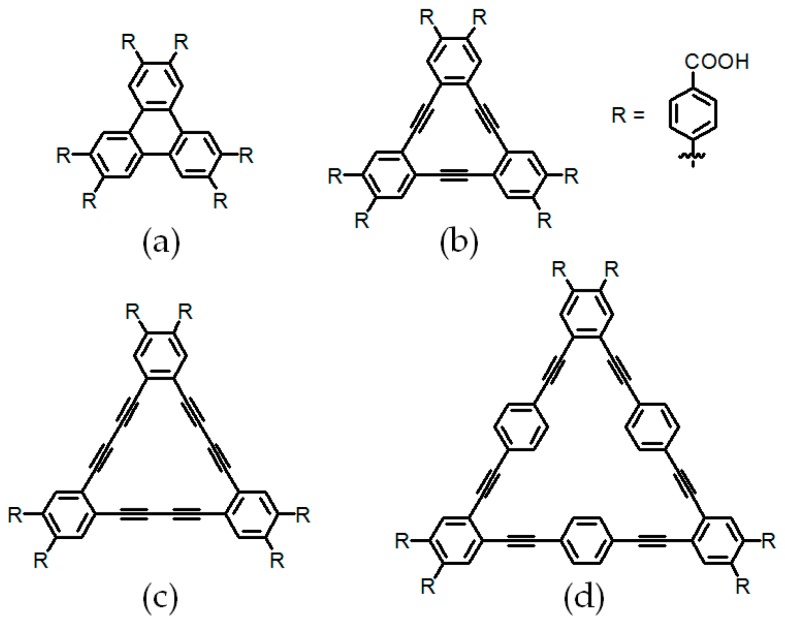

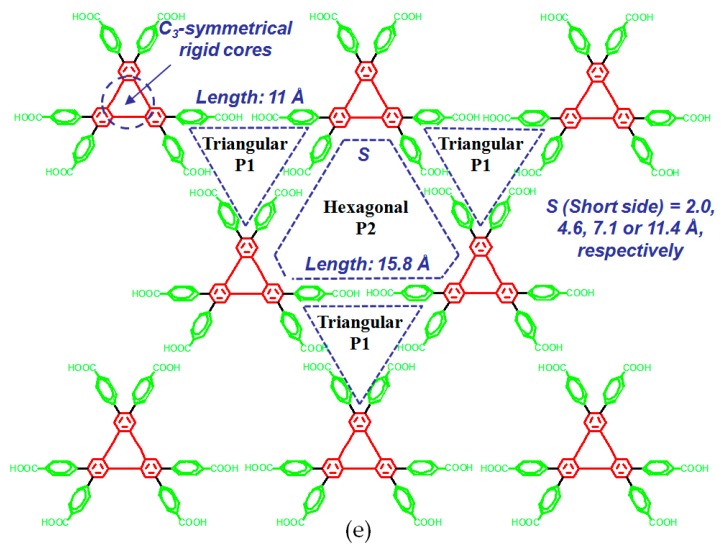
Structures of building motif **6a** (**a**); **6b** (**b**); **6c** (**c**); and **6d** (**d**); (**e**) Graphic representation of hydrogen bonded hexagonal sheets.

The complexation and porosity properties of *C*_3_-symmetric carboxylic acid analogues **6a**–**6c** (see Figure 7) were also examined [34,35]. Through rational design, three 4,4′-dicarboxy-*o*-terphenyl subunits were connected together via Sonogashira or Suzuki-Miyaura coupling reactions. In accordance with the crystalline geometry of 1,3,5-benzenetricarboxylic acid, these building motifs (see Figure 7) self-assemble into HOFs with ordered hexagonal networks, which display diverse porosities range from 38% to 59%. In crystal packing structures, *C*_3_-symmetric carboxylic acid motifs (see Figure 7) firstly associate with six adjacent motifs to result in the formation of porous hexagonal hydrogen bonded sheets, thereby all these sheets further extended to more intricate three dimensional networks via efficient crystal packing. In crystalline HOFs (see Figure 7e), two types of porous geometries (P1 and P2) were confirmed to result from hydrogen bonding motifs, as verified by systematically crystallography and theoretical computational studies. Interestingly, the geometrical changes in *C*_3_-symmetric rigid cores indeed do not interfere with the size and shape of P1, whose triangular length in all cases were determined to be 11 Å. In sharp contrast, the size of hexagonal P2 is closely correlated with the length of triangular cores in building motifs (Length × S: 15.8 × 2.0 Å^2^ for **H6a**, 15.8 × 4.6 Å^2^ for **H6b**, 15.8 × 7.1 Å^2^ for **H6c**, and 15.8 × 11.4 Å^2^ for **H6d**), thereby affecting porous performances of **HOFs** (see Figure 7e). Depending on combination of non-covalent bindings, including C–H⋯π, C–H⋯O or π⋯π stacking interactions, two-dimensional hydrogen bonding sheets finally transform into three-dimensional topologies. In terms of gas absorption, activated **HOFs** containing **6a** or **6b** exhibited promising performances in CO_2_ gas uptake (BET surface is 788 m^2^/g for **H6a** and 557 m^2^/g for **H6b**) due to crystallinity maintenance and permanent porosity. Evidently, the increasing in pore sizes does not always accompanying with significant improvement in porosity properties.

**Figure 8:**
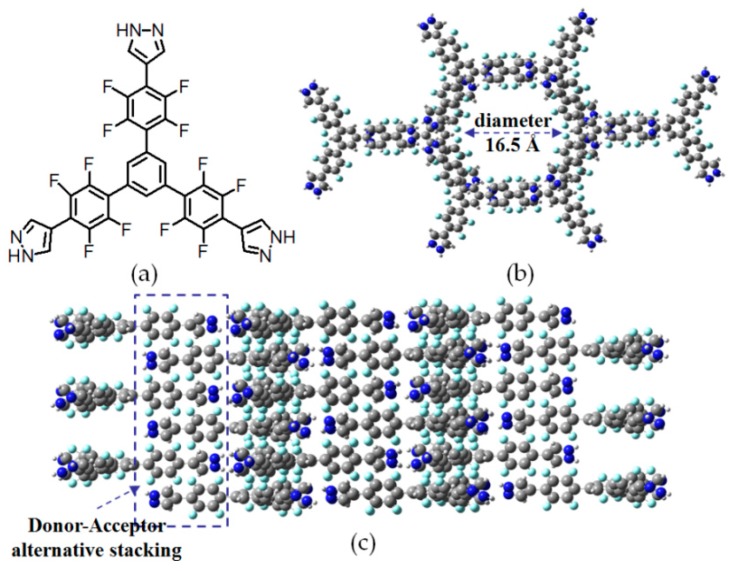
(**a**) Structure of building motif **7**; (**b**,**c**) Hexagonal channels and alternative crystal stacking of **H7**.

Besides the aforementioned *C*_3_-symmetric motifs possessing multiple carboxylic acid groups, crystalline porous HOFs, grown from *C*_3_-symmetric motif **7** (see Figure 8a), not only exhibited promising performance in CO_2_ uptake, but also displayed unique advantages to stabilize some specific aliphatic guests, including fluorocarbons (perfluorohexane), hydrocarbons (cyclohexane or hexane), or chlorofluorocarbons (CFCs such as Cl_2_FC–CClF_2_) [36].

Instead of conventional carboxylic acid groups, three pyrazole units were deliberately integrated into motif **7** to act as the role of hydrogen bonding sites. More interestingly, pyrazole moieties were attached to benzene core via electron-deficient 2,3,5,6-tetrasubstituted fluorobenzene groups. It is pointed out that pyrazole units in motif **7** indeed play two crucial roles in resultant HOF. Firstly, each pyrazole subunit interacts with two neighboring pyrazoles via strong N–H⋯N hydrogen bonds. Secondly, donor–acceptor interactions are responsible for alternative π⋯π stacking between electron-rich pyrazole planes and electron-deficient fluorobenzene groups (see Figure 8c). In crystalline **H7**, characteristic hexagonal interconnected channels (see Figure 8) were observed and the average diameter of channels was calculated to be approximately 16.5 Å. In addition, the BET surface was determined to be 1159 m^2^/g even though the porosity of **H7** was moderate (51%).

### 2.3. Amide or Urea Groups as Hydrogen-Bonded Motifs for HOFs

**Figure 9:**
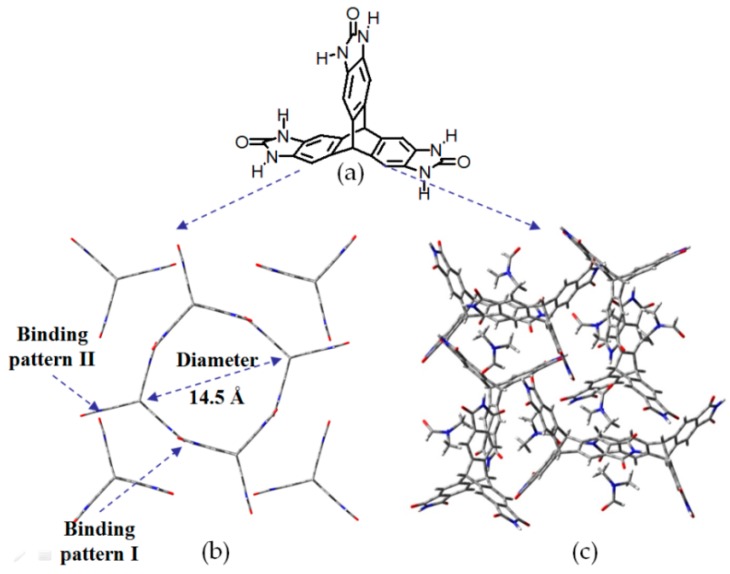
(**a**) Structure of the building motif of **H8**; (**b**) The Top view of porous network of **H8** (grown from acetone/DMSO); (**c**) Crystalline porous network of TTBI grown from DMF/acetone.

Self-complexation of amide groups has been considered as another tool to construct porous HOFs. For instance, Mastalerz designed and synthesized porous **H8** with excellent gas absorption performance and extremely high BET surface area [37]. This porous HOF was obtained from acetone/DMSO by self-assembly of *D*_3h_ symmetric building motif triptycene-trisbenzimidazolone (TTBI) (see Figure 9a). Two types of hydrogen-bonding patterns were observed, namely pattern I and II. Pattern I plays critical role in defining porous channels, which show an amazing average diameter of 14.5 Å (see Figure 9b). However, pattern II is mainly responsible for the connection of channels via residual hydrogen bonding sites of TTBI, which do not participate in the formations of channels. Thanks to the extremely high BET surface area (2796 m^2^/g), the crystalline HOF exhibits promising gas sorption behaviors. In detail, when this crystalline HOF was exposed to hydrogen gas at 77 K under a pressure of 1 bar, the activated HOF was capable of taking up 2.2 wt % hydrogen gas. Moreover, the absorption capacity of CO_2_ was also encouraging. The activated HOF can absorb 15.9 wt % CO_2_ under testing conditions of 273 K and 1 bar. Apart from large BET surface area, the enhanced CO_2_ absorption can be partially attributed to the strong interaction between interior benzimidazolone framework and negative quadrupole moment of CO_2_. In sharp contrast, the HOF exhibits relatively poor CH_4_ absorption performance, probably due to the rather weak binding affinity between intercalated CH_4_ and inferior surface of channels.

However, the honeycomb-shaped **H8** was not obtained as anticipated when there exist evident differences in composition of solvents during crystal growth. The TTBI building motif, which was crystallized from DMF/acetone instead of acetone/DMSO, merely assembles into crystals with highly disordered topology (see Figure 9c) rather than other reported highly ordered channels [38]. The sharp contrast in porous crystal structure unambiguously indicates that solvent significantly affects the crystallization process as well as porosity of HOFs.

**Figure 10:**
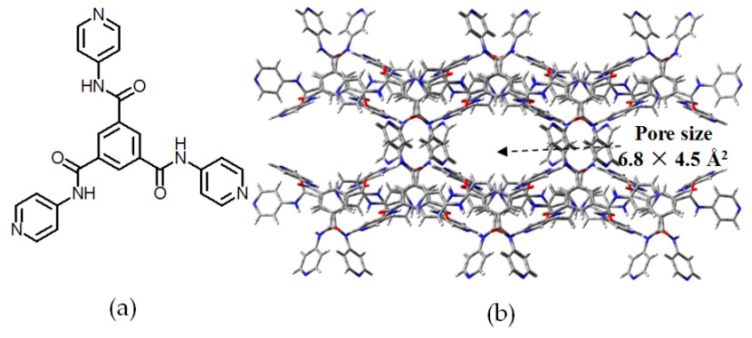
(**a**) Structure of the building motif of **H9**; (**b**) Crystalline geometries of porous **H9**.

Porous **H9** was obtained from hydrogen bonding self-complexation of multiamide monomer *N*_1_,*N*_3_,*N*_5_-tris(pyridin-4-yl)benzene-1,3,5-tricarboxamide (TPBTC) (see Figure 10a) [39]. Notably, Meijer and coworkers studied a collection of polymeric aggregates assembled from multiamide-based *C*_3_-symmetrical aromatic monomers [40,41]. However, these aggregates have no porous properties, since monomers closely stacked with each other via combination of amide hydrogen bonding interactions and π-π stacking. In the case of TPBTC, the N atoms in three pyridine subunits act as hydrogen-bond acceptors, whilst the H atoms in amides act as donors. Although the TPBTC building motif (see Figure 10a) displays limited binding sites and weak binding strength, but the resulting **HOF** (see Figure 10b) apparently shows amazing thermodynamic stability. Moreover, the desolvated HOF exhibits unexpected absorption affinities toward CO_2_ and benzene at ambient temperature. These properties were demonstrated by the X-ray diffraction (XRD) geometry. In the crystal structure, the average size of pores is 6.8 × 4.5 Å^2^, and the accessible volume for solvent guests is ~24%, which is evidently inferior to those of HOFs obtained from DAT derivative building motifs. On the basis of the isotherm method, gas (N_2_, CO_2_, and H_2_) absorption capacities were investigated. Unexpectedly, only CO_2_ exhibits nominal affinity properties, which can be reasonably attributed to inductive effect between CO_2_ and the HOF framework owing to the negative quadrupole moment. Furthermore, the benzene affinity was determined by ^13^C-NMR spectra and TGA. After addition of benzene, a resonance peak (128.3 ppm) appeared in ^13^C-NMR spectrum, whereas the accommodated benzene molecules were able to be completely removed from the HOF framework when heated to 355 °C.

**Figure 11:**
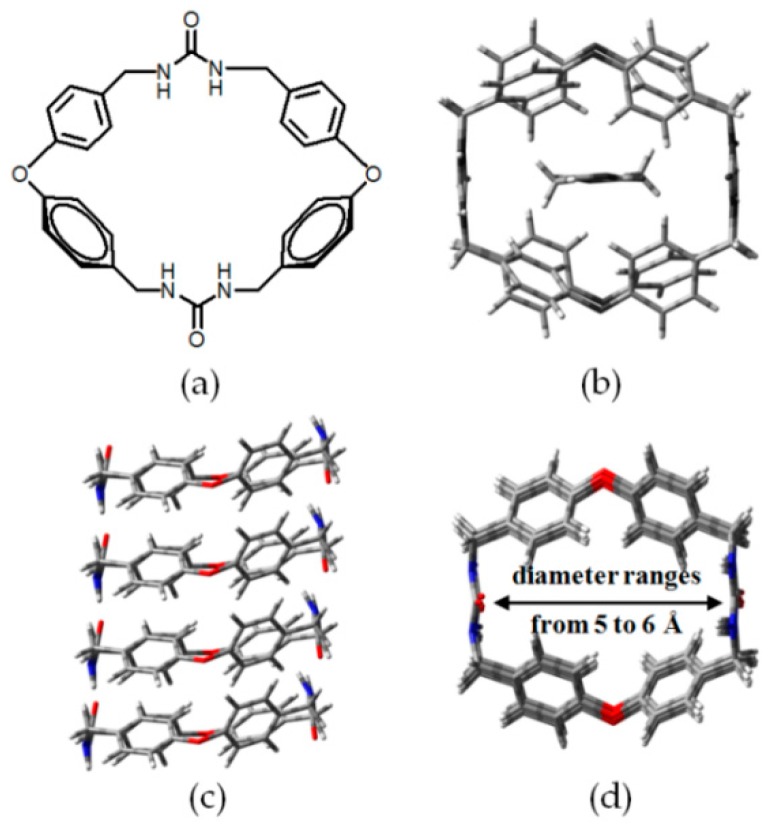
(**a**) Structure of the building motif of **H10**; (**b**) Top view of the crystalline porous network of **H10** with two acetic acid (CH_3_COOH) molecules as the guests; (**c**,**d**) Side and top view of the crystalline network of **H10**.

The macrocyclic monomer (see Figure 11a) bearing two urea groups can efficiently form porous channels (see Figure 11c) via N–H⋯O stacking. The porous HOF exhibited satisfactory CO_2_ uptake performance and unique selectivity when treated with aliphatic carboxylic acids [42,43]. At 195 K, the CO_2_ absorption capacity of **H10** was determined to be approximately 71.5 cm^3^/g. Theoretical computations based on NLDFT and PALTON clearly demonstrated that the pore diameter in **H10** mainly ranged from 5 to 6 Å (see Figure 11d) and the porosity was estimated to be about 13.7%. Interestingly, the HOF is capable of binding to linear aliphatic carboxylic acids (see Figure 11b) with different molar fractions, depending on the molecular sizes of acid guests. It is worthy of note that, for aliphatic carboxylic acids with longer chain (C ≥ 7), no evident association was observed [43].

### 2.4. Macrocyclic Receptors as Hydrogen-Bonded Motifs for HOFs

Despite the fact organic frameworks based on macrocyclic compounds are generally classified into supramolecular organic framework (SOFs) category, noncovalent hydrogen bonding interactions are intrinsic driving forces in the fabrication of some SOF materials. Therefore, we still consider these hydrogen-bonded SOFs as classical examples of HOFs in this review.

**Figure 12:**
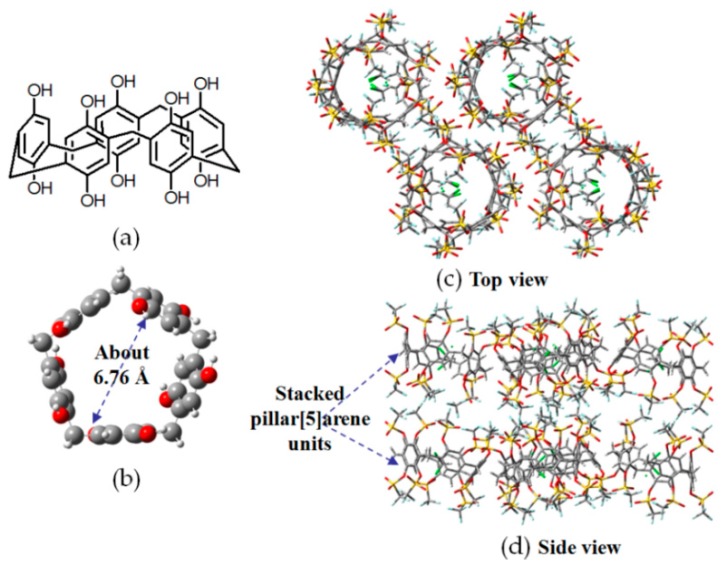
(**a**) Structure of the building motif of **H11**; (**b**) Pentagonal cavity of pillar[5]arene; (**c**,**d**) Top view and side view of crystalline network of **H11**.

Pillarenes, which have been widely used in the constructions of novel pseudorotaxanes or versatile stimuli-responsive supramolecular polymers [44,45,46], are a type of symmetric pillar-shaped macrocyclic compounds with rigid and π-rich inner cavities. This has inspired researchers toutilizepillar[5]arene as gas absorption and separation materials. The pentagon-shaped pillar[5]arene (see Figure 12a,b) forms a three-dimensional (3D) open network (see Figure 12c) through hydrogen bonds formed by the outer OH groups in the top and bottom of the cavity rim(see Figure 12d) [47]. Each pillar[5]arene associates with identical motifs by employing O–H⋯O bonds. The channels, which are responsible for gas storage, have an average diameter of ~6.76 Å. As shown in Figure 12d, each pillar[5]arene monomer closely stacks with adjacent motifs by robust O–H⋯O interactions to further extend channels, thus stabilizing the porous 3D crystalline framework. Experiments on CO_2_, H_2_, and N_2_absorption (298 K and 1 atm) unambiguously demonstrated that perhydroxyl-pillar[5]arene had high CO_2_ sorption capacity (88 mg/g). Notably, the absorption ratio of CO_2_/N_2_ and CO_2_/CH_4_ were 339 and 375, respectively, indicating that the HOF displays highly affinity toward CO_2_. Moreover, the BET surface area and the total pore volume were estimated to be 97 m^2^/g and 0.34 cm^3^/g, respectively.

**Figure 13:**
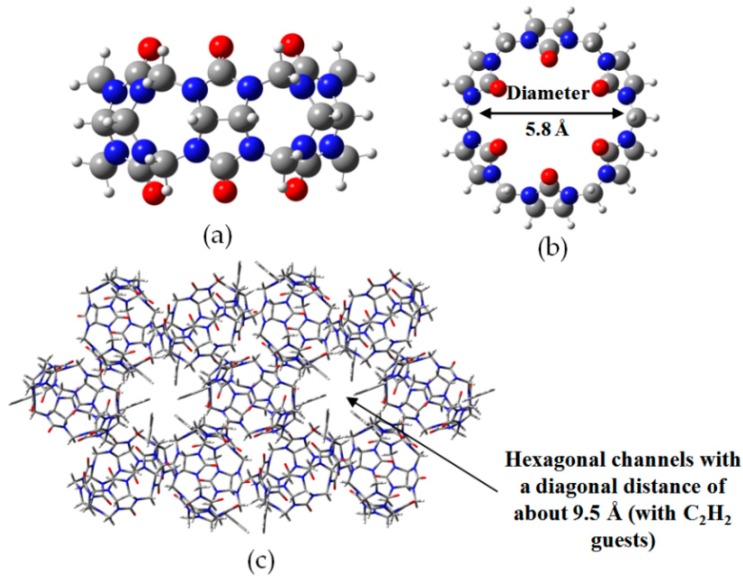
Side (**a**) and top view (**b**) of CB[6]; (**c**) The porous crystalline network of **H12**.

Analogously, HOF self-assembled from cucurbit[6]uril (CB[6]) (see Figure 13a,b) and stabilized by combination of noncovalent C–H⋯O hydrogen-bonding interactions and Van der Waals forces, exhibit promising thermal stability and excellent C_2_H_2_ absorption performance [48] which indicate the great potential of cavity compounds in forming HOFs. As shown in Figure 13c, the crystal structure of **H12** demonstrated that every six CB[6] motifs are connected together by multiple hydrogen bonds and exterior noncovalent contacts of CB[6] surfaces to result in the formations of hexagonal channels, which provided additional binding sites for intercalated C_2_H_2_. The absorption isotherm experiments well-illustrate the C_2_H_2_ absorption property of **H12**. Notably, the C_2_H_2_ gas uptaking capacities for activated **H12** were estimated to be approximately 11 wt % (196 K) and 6.1 wt % (298 K), respectively. The exceptional high absorption ability was also confirmed by XRD. According to XRD characterization, the volume of space in crystalline **H12** was calculated to be 1557.8 Å^3^, which precisely suitable to accommodate 4 acetylene molecules at most.

**Figure 14:**
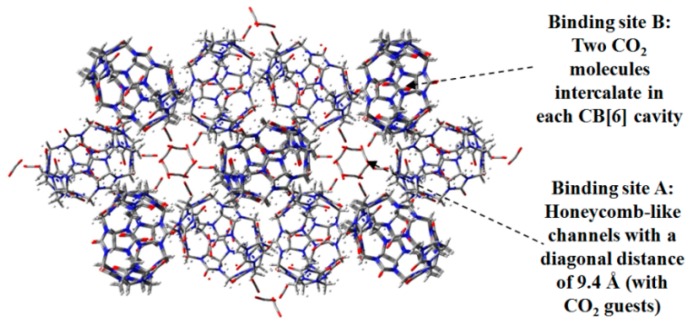
X-ray diffraction geometry of crystalline porous **H13** with CO_2_ as guest.

Additionally, Kim further investigated the potential of the CB[6] motif in the selective absorption of CO_2_, CO, and CH_4_ [49]. The CB[6]-based **H13** exhibits a gas absorption capacity of 45 cm^3^/g under conditions of 298 K and 1 bar. Most importantly, the HOF displays nominal selectivity between CO and CO_2_. Under the experimental conditions (196 K and 1 bar), the HOF showed a maximum CO_2_ absorption capacity of 97 cm^3^/g, whereas the material was inert when exposed to CO gas. The XRD analysis of CO_2_-absorbed crystalline **H13** (see Figure 14) illustrates well why the porous material possesses an extremely high binding affinity toward CO_2_. The structure has two important binding sites. Binding site A (see Figure 14) is mainly located in the exterior of CB[6]cavity, and CO_2_ molecules are captured by the hydrogen-bonding interactions between CH groups in CB[6] and carbonyl O atoms in CO_2_ with an average bond length of 2.575 Å. Another binding pattern (see Figure 14) is ascribed to the quadrupole interactions between CO_2_ molecules and inner π-conjugated surface of CB[6]. Interestingly, the conformation of CB[6] and the portals of cavities are blocked by the adjacent building motifs to prevent the desorption of gas.

It has to be noted that the cavity sizes of pillarenes or cucurbituril can be tuned by varying the number of phenol or glycoluril subunits in macrocyclic geometries. Considering that the cavities in macrocycles are confirmed to participate in non-covalent binding of gas, we speculate that such changes in cavity size and shape may have significantly influences on the binding manners of intercalated gas molecules, thereby affecting absorption capabilities or porous properties of HOFs. Probably due to tedious and great difficulties in synthesis of building motifs possessing extended pillar[*n*]enes (*n* > 5) or cucurbit[*n*]uril (*n* > 6) macrocycles, they are indeed seldom reported in the construction of functionalized HOF materials. Apart from the aforementioned supramolecular hosts, other types of macrocycles, including derivatives of calix[*n*]arenes, are also capable of forming versatile HOF materials with excellent performances in guest release [50,51] or gas storage [52].

### 2.5. Linear Dipeptide as Hydrogen-Bonded Building Motifs for HOFs

Amino acids have emerged as another type of promising building motifs for hydrogen-bonded microporous materials. As compared to other building motif categories discussed in this review, dipeptide arrays indeed absence of rigid scaffold, which may partially illustrate the relatively poor performances in porous properties. Commonplace dipeptide arrays, including Val–Ala, Phe–Phe, Leu–Ser, and Ile–Ile have been investigated in corresponding articles. Dipeptides containing NH_3_^+^ and COO^−^ groups can pack together in head-to-tail self-assembly patterns by combination of hydrogen bonding interactions and Van der Waals forces to result in the formations of hydrophobic chiral channels or nanotubes in HOFs. In term of gas selectivity, recent research has revealed that the size complementarity between intercalated guests and pore diameter played crucial roles [53,54,55]. Quite different from macrocyclic hosts in the aforementioned HOF materials, the size of hydrophobic pores can be regulated by changing the composition of dipeptide sequences (see Table 2) [56].

**Figure 15:**
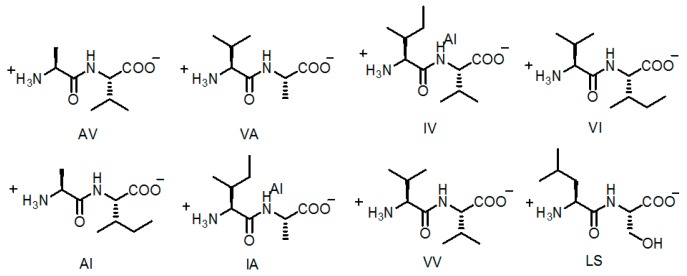
Structures of the building motifs of **H14a**–**14h**.

Soldatov investigated eight dipeptide-based hydrogen-bonding assembled crystals **H14a**–**14h** (see Figure 15) and further examined their Xe absorption performance [53]. Not only did dipeptides assemble together by hydrogen bonding, but also micropores in such HOFs were extended to form one-dimensional open channel-like nanotubes. With larger channel diameter and higher porosity, **H14a** and **H14b** showed excellent affinities toward Xe gas.

**Figure 16:**
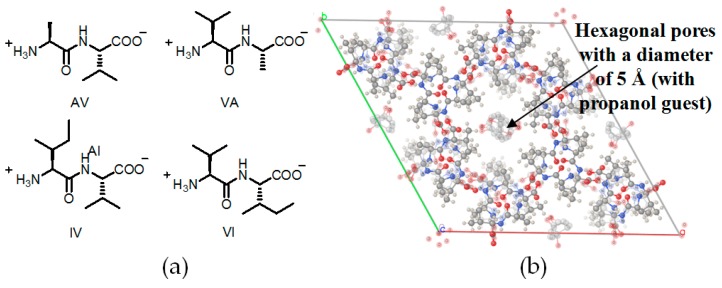
(**a**) Structure of building motifs of **15a** (AV: L-Ala–L-Val), **15b** (VA: L-Val–L-Ala), **15c** (IV: L-Iso–L-Val), and **15d** (VI: L-Val–L-Iso); (**b**) The hexagonal shaped pore in crystalline **H15a** (crystal grown from 2-propanol) [55].

Dipeptides with biological affinity were utilized in the constructions of crystalline **H15a**–**15d** (see Figure 16a for building motifs **15a**–**15d**) with chiral hydrophobic pores (see Figure 16b) and further investigate their gas absorption properties [54]. As shown in Figure 16a, four types of linear dipeptide sequences self-assembled into supramolecular aggregates via hydrogen-bonding interactions, thereby leading to the formations of hydrophobic micropores with different diameters, as observed in the aforementioned example. Interestingly, by varying the micropore diameter, the gas selectivity of such HOFs can be tuned in controllable manners. In detail, when **H15d** was utilized in absorption isotherm experiments, the molar absorption ratio between CO_2_/CH_4_ was calculated to range from 3.5 to 5, whereas the corresponding values of **H15a** and **H15b** merely ranged from 2 to 2.5 under the same conditions. The evident drop in selectivity can be ascribed to the evident decrease in micropore diameter. In the case of **H15a**, the pore diameter of crystal was 5.0 Å. However, the pore sizes of **H15b**–**15d** were calculated to be approximately 4.7, 3.9, and 3.7 Å, respectively. Due to the relatively small pore size in **H15c** and **H15d**, both of them are capable of absorbing hydrogen gas efficiently. The designing protocol provide us with an emerging strategy to tune and manipulate the diameter of pores via the self-assembly of various peptides.

### 2.6. Pyridone or UPy Moieties as Hydrogen-Bonded Motifs for HOFs

In order to construct porous HOFs more efficiently, it is urgent to design sticky sides with lower cost and facile synthesis if porous HOFs are going to be widely applied in industrial or medical fields such as gas storage, isolation or drug delivery. As discussed above, B(OH)_2_-substituted tetraphenylmethane or tetraphenylsilane building motifs achieve prominent 60% and 64% porosity, respectively, but it is relatively hard to integrate multiple B(OH)_2_ groups into a rigid backbone at one time without the assistance of expensive, dangerous, and toxic reagents such as *n*-BuLi and *t*-BuLi. As ideal sticky sides of porous HOF materials, it is crucial to achieve high porosity at a reasonable cost. Hence, scientists still need to seek for more accessible sticky sides.

**Figure 17:**
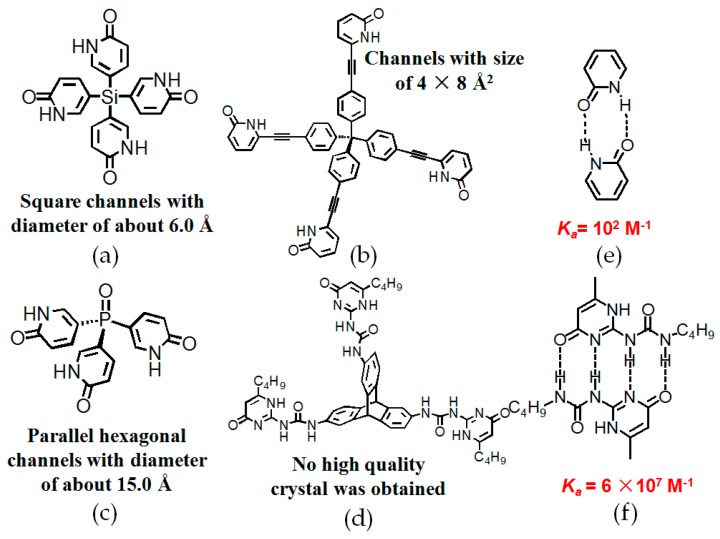
Structures of building motif **16** (**a**); **18** (**b**); **17** (**c**); and **19** (**d**); (**e**) dimeric 2-pyridone array; (**f**) dimeric UPy array.

In the pioneering stage of hydrogen bonding arrays, pyridone moieties were investigated by Wuest, etc. [57,58,59] and this is really a good example to illustrate how hydrogen-bonded arrays have significantly impacts on the rapid development of porous HOFs. Initially, they found that dimerization strength of 2-pyridone was determined to be approximately 10^2^ M^−1^ (see Figure 17e), indicating that self-complexation affinity was rather weak [57]. By utilizing coupling reactions between pyridone moieties, the sticky side can be conveniently converted into self-aggregated polymeric homoditopic monomers [58,59]. As monomer concentration is gradually increased, the topologies of the resultant supramolecular polymers undergo a clear transition from cyclic to linear polymeric species [58,59]. Motivated by this success, the pyridone moiety was then integrated into building motifs **16**–**18** (see Figure 17a–c) as sticky sites to create porous HOFs. Unexpectedly, the porosity of **H16** was calculated to be an impressive 60%, which apparently exceeded most previously reported HOFs and this progress could be unambiguously regarded as a meaningful milestone in the development of porous HOFs. Later on, they designed two counterparts **17** and **18**, which contained different elaborately designed backbones [60,61]. In detail, they endeavored to investigate the influence of polar P=O bonds on porosity. As planned, the trigonal building motif **17** associated together to afford sheets with hexagonal micropores. Meanwhile, the hydrogen bonding and dipolar effects of P=O bonds worked in a synergistic manner to facilitate stacking of adjacent sheets and thus, giving rise to the generation of three-dimensional supramolecular networks. Nevertheless, **H17** merely exhibited moderately 50% porosity in its crystalline architecture, which was obviously inferior to that of **H16** [60]. Apart from the strategy of introducing dipole effects to direct self-assembly, Wuest also designed an enlarged building motif containing the tetrakis(4-alkynylphenyl)-methane backbone, assuming that this rational design would be beneficial to enlarge pore size. However, as verified by XRD studies of **H18**, only 24% of the space volume was available for accommodation of guests in the resulting diamondoid network due to undesirable hydrogen-bonded associations between the pyridone moieties and CH_3_CH_2_COOH [61].

Nevertheless, the weak binding strength (*K_a_* = 10^2^ M^−1^ in CHCl_3_) between self-complementary 2-pyridone assemblies may pose threats to the robustness and permanent porosity of HOFs. To the best of our knowledge, the 2-ureido-4[[1*H*]-pyrimidinone (UPy) moiety, which is regarded as a milestone in the development of robust hydrogen bonding array, exhibits the strongest binding affinity (*K_a_* = 6 × 10^7^ M^−1^ in CHCl_3_) in the formation of self-complementary dimer (see Figure 17f). Considering that the dimer is prone to compatibility with other sorts of host/guest assembly systems, the UPy moiety has been widely utilized in the construction and fabrication of stimuli-responsive supramolecular polymers [62,63] since it was first reported by Meijer and coworkers in 1997 [64]. Nevertheless, HOFs containing UPy subunits as sticky sides were rarely reported, even though the hydrogen-bonded UPy array possesses so many advantages. Recently, Han and co-workers designed a novel motif **19** (see Figure 17d) containing three UPy moieties as terminal sticky sides and a rigid triptycene scaffold as central linker [65]. As compared to DAT-based HOFs, it was confirmed that it was more difficult to obtain a crystalline framework for **H19**. Isotherm absorption experiments demonstrated that the BET surface area of **H19** was estimated to be 32 cm^2^/g, which is significantly lower than that of the aforementioned examples. Although the BET surface of this HOF was less appealing, it displayed very impressive affinity towards CO_2_ (selectivity up to 96) when it was exposed to CO_2_/N_2_ at 273 K [65]. In contrast, the triptycenetrisbenzimidazolone (TTBI) motif possessing the identical triptycene core assembly as **H8** has an amazing BET surface area of 2796 m^2^/g, which is remarkably higher than that of **H19**. Unambiguously, UPy units have much stronger binding propensity with respect to that of amide groups. Since the UPy moiety fails to maintain a rigid scaffold due to the presence of the flexible urea groups, thereby the building motif may be incapable of maintaining the corresponding porous framework structure. In related work, it was found that elimination of flexible urea linkers facilitated self-assembly of porous HOFs to some extent when triptycene was directly attached to dimeric B(OH)_2_ or pyridone via C–C bonds, but the porosities of the resulting HOFs assembled from triptycene-based tripyridone or triboronic motifs were even less appealing (less than 10%) [66]. From our perspective, it is really hard to say which factor (rigidity of the building motif or self-complementary binding strength) plays a more decisive role in the enhancement of HOF materials, but we may draw a conclusion from the comparison: it is crucial for us to take both factors into account before everything is known for sure.

### 2.7. DAT or DAP Moieties as Hydrogen-Bonded Motifs for HOFs

**Figure 18:**
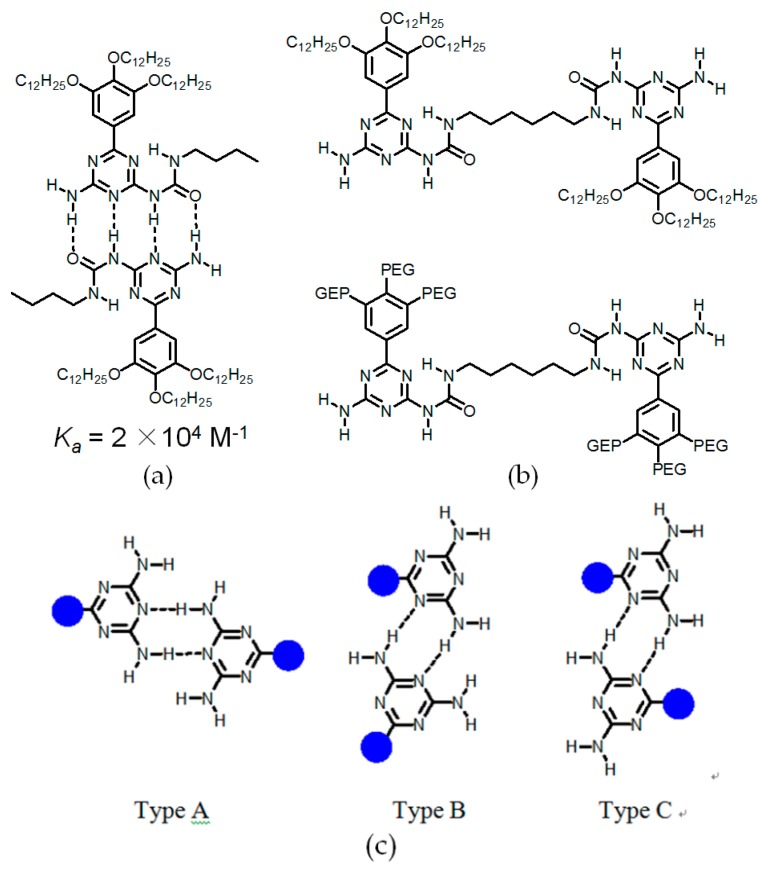
(**a**) Representative example of DAT based hydrogen bonding array; (**b**) supramolecular polymeric monomers containing DAT moieties; (**c**) feasible hydrogen-bonding assembled patterns between DAT–DAT motifs.

As a versatile self-assembly motif, 2,4-diamino-1,3,5-triazine (DAT) has attracted much attention. Meijer was the first chemist to explore the hydrogen bonding manners of DAT-based self-complementary arrays. By rational designing strategies, these hydrogen-bonded arrays show strong binding affinities in apolar media (see Figure 18a) [67,68]. Accordingly, various supramolecular monomers (see Figure 18b) were designed and prepared to construct functionalized liquid crystal materials [67,68]. In analogy to pyridone and UPy moieties, DAT is also deliberately integrated into the construction of HOFs. Quite different from the above mentioned DAT-based arrays, the DAT unit is capable of adopting three feasible binding patterns to form dimeric species (see Figure 18c) in HOF materials. The self-assembly of DAT favors type A the most, since the two bulky residual groups of building motifs are far away from each other. To the best of our knowledge, Wuest and coworkers reported a collection of pioneering studies on DAT units for constructing porous HOFs by supramolecular crystal engineering [69].

#### 2.7.1. Tetrahedral DAT-Based Building Blocks

**Figure 19:**
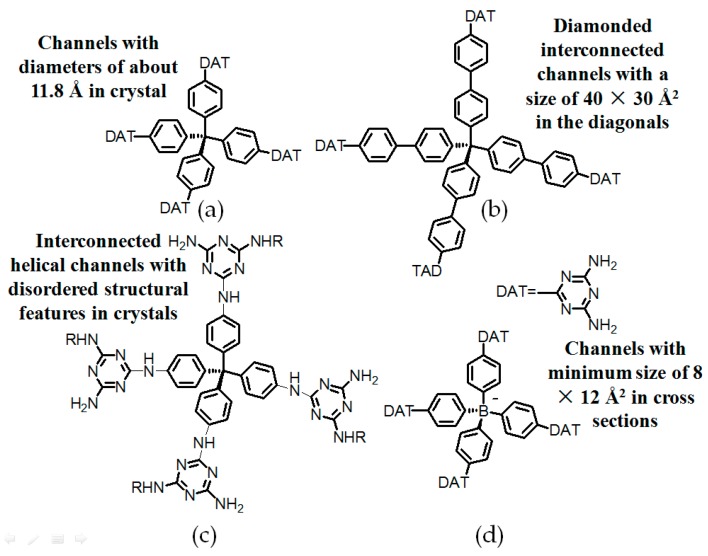
(**a**) Structure of the building motif **20**; (**b**) Structure of motif **21**; (**c**) Structures of motif **22a** (R = H), **22b** (R = CH_2_CH_2_OCOCH_3_), and **22c** (R = CH_2_CH_2_OH); (**d**) Structure of motif **23**.

**Figure 20:**
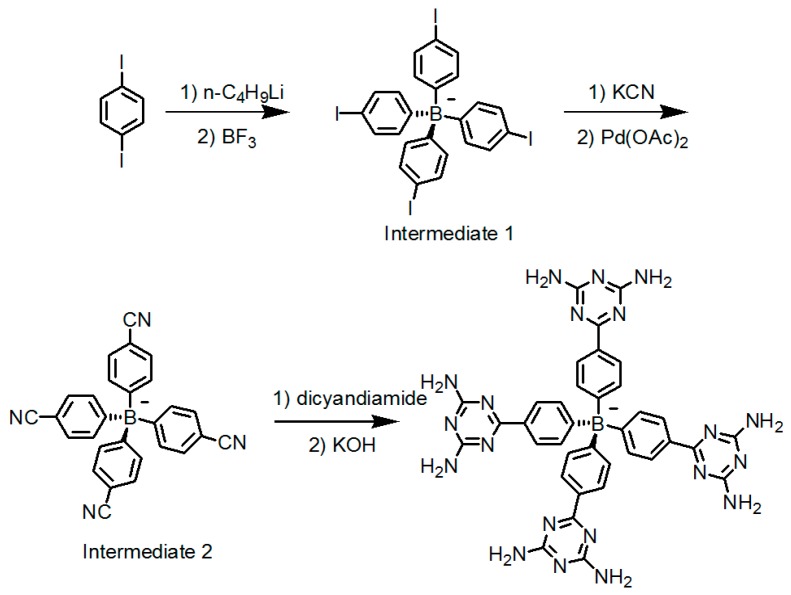
Synthesis route of negative charge tetrahedral building motif **23**.

**Figure 21:**
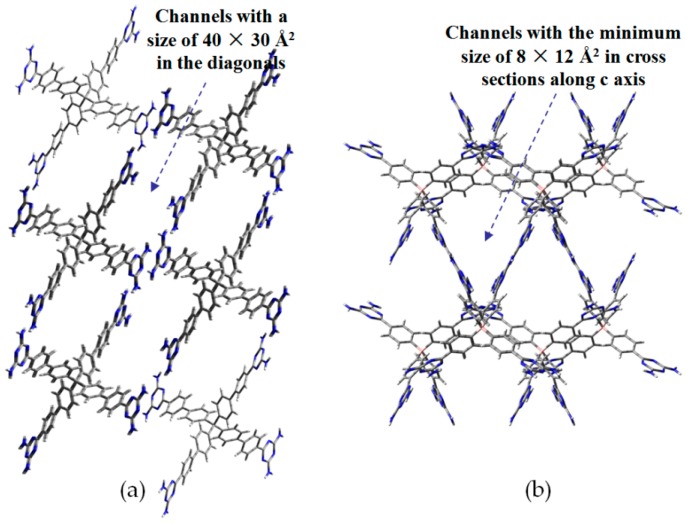
Crystalline porous network of **H21** (**a**) and **H23** (**b**).

Considering that combination of B(OH)_2_ and a tetrahedral backbone endowed HOFs with desired diamondoid network structures and outstanding porosities, thereby the representative tetrakis(phenyl)methane scaffold was integrated into the building motif (see Figure 19a) [70]. As witnessed by crystal structure, the type B hydrogen bonding model is predominately found in **H20** and each DAT moiety associates with two adjacent building motifs. In addition, this HOF contains interconnected channels in its microstructure and its porosity was calculated to be approximately 45%. The extraction of solvent guests from interconnected channels did not result in the eventual disruption of the crystalline geometry and merely a slight crystal contraction was observed [70]. This unique characteristic was frequently demonstrated in MOFs such as zeolites but rarely observed in HOFs owing to their relatively brittle frameworks and cohesion abilities. On this basis, Chen presumed that insertion of multiple phenyl spacers between tetrakis(phenyl)methane and DAT moieties (see Figure 19b) would give rise to enlarged internal voids, which could definitely facilitate the inclusion of guests. Nevertheless, only a moderate 42.5% porosity was observed in the resultant **H21** (see Figure 19b) although it exhibited desirable selectivity in the isolation of C_2_H_4_/C_2_H_6_ [71]. Since binding motifs **20** and **21** are intrinsically deficient in flexibilities, both structures maybe incapable of adjusting themselves to form hydrogen-bonded supramolecular networks. In a related work, Wuest designed a tetrakis(4-aminophenyl)methane backbone which permitted all DAT groups to be linked with the tetrakis(phenyl)methane motif via more flexible NH spacers [72]. By rational design, the NH_2_ groups in this backbone can be substituted by DAT derivatives to afford analogues **22a**, **22b**, and **22c** (see Figure 19c), respectively. Interestingly, XRD studies of single crystals demonstrated that merely the NH_2_ groups and NH spacers in these analogues were evidently involved in the formation of NH-N hydrogen bonds, which was quite different from the case of DAT-DAT dimers. However, both HOFs still exhibited durable resistance to loss of crystallinities and porosities which for **H22b** and **H22c** were determined to be approximately 40% and 50%, respectively, suggesting that alkyl chain substitution of NH_2_ groups does not interfere with inclusion of external guests. From the porosity values, we speculate that this kind of HOF may already approach the theoretical porosity limitation, and thereby it is meaningless to persist in further modification of tetrahedral tetrakis(phenyl)methane backbone. Wuest found that the combination of hydrogen bonding and charge interactions might be another accessible method for achieving high porosity. In their structures, four DAT subunits were connected to an anionic B(PPh)_4_^−^ scaffold [73]. As can be seen (see Figure 20), 1,4-diiodobenzene initially reacts with boron trifluoride ether complex in the presence of *n*-BuLi to afford tetrabromide intermediate **1**. After a nucleophilic aromatic substitution (S_N_Ar) reaction, intermediate **2** is synthesized in decent yield. Finally, intermediate **2** is treated with dicyandiamide and converted into anionic building motif **23** (see Figure 19d) with 61% overall yield. Quite different from all the aforementioned neutral analogues, each building motif in **H23** merely interacted with six adjacent motifs by adopting all feasible self-complementary conformations of DAT dimers to afford a supramolecular network. It is noteworthy that for **H23** (see Figure 21b for crystal structure), the remarkable 74% porosity is evidently higher than that of neutral HOF analogues.

Apart from systematic studies on rigid tetrakis(phenyl)methane scaffold, other types of porous HOFs possessing flexible 2,4,8,10-tetraoxaspiro [5,5]undecanes, pentaerythrityltetraphenyl ether, or dipentaerythritylhexaphenyl ether as basic building skeletons were also carefully investigated.

**Figure 22:**
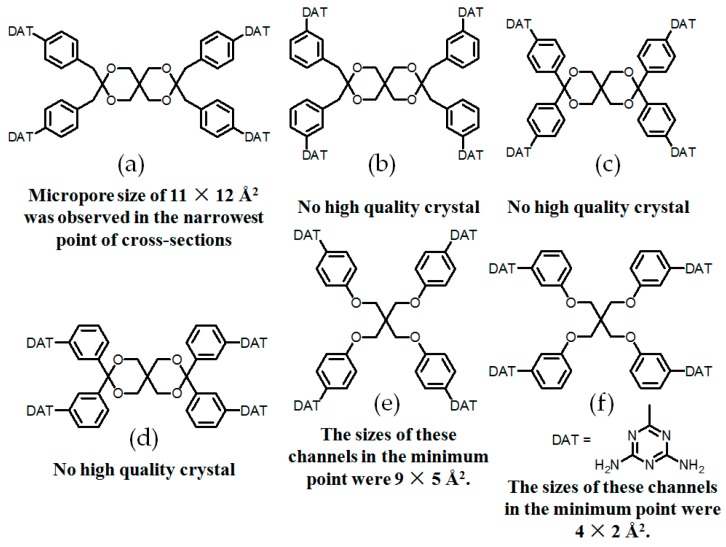
(**a**) Structure of building motif **24a**; (**b**) Structure of building motif **24b**; (**c**) Structure of building motif **24c**; (**d**) Structure of building motif **24d**; (**e**) Structure of building motif **25a**; (**f**) Structure of building motif **25b**.

**Figure 23:**
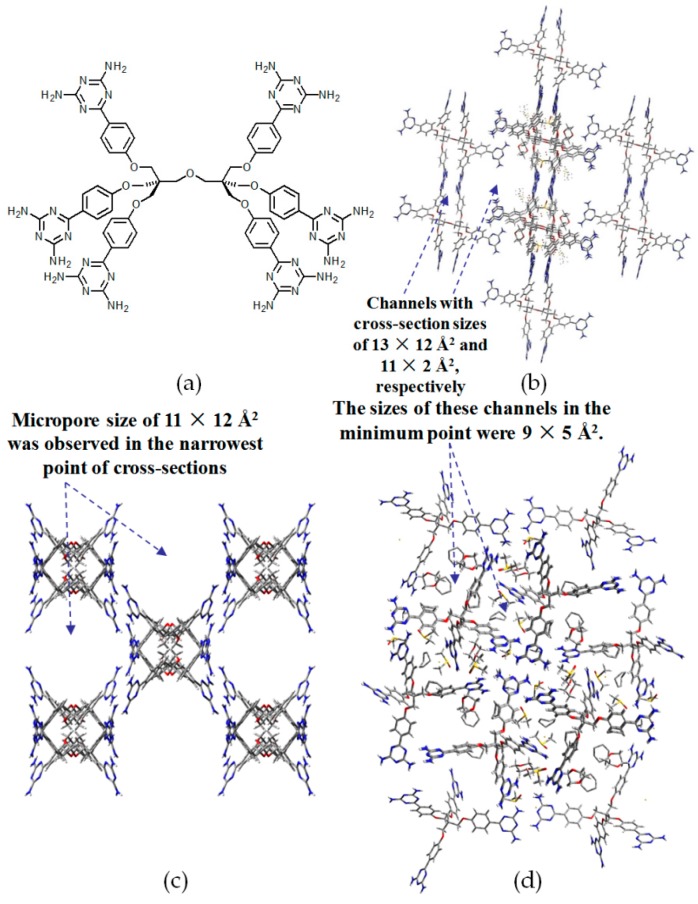
(**a**) Structure of building motif **26**; (**b**) Crystalline network of **H26**; (**c**,**d**) Top views of **H24a** and **H25a** (with solvent guests), respectively.

In 2003, Wuest reported four classical **H24a**–**24d** (see Figure 22a–d), whose building motifs are derived from the flexible tetrasubstituted-2,4,8,10-tetraoxaspiro[5,5]undecane scaffold [74]. However, X-ray diffraction results of crystals revealed that both type A and C binding models participated in self-assembly of DAT moieties and crystalline **H24a** (see Figure 23c for HOF geometry) grown from DMF/toluene had a porous network with about 60% accessible volume for guests, whereas the supramolecular networks of **H24b**–**24d** were not reported yet due to failures in obtaining high quality single crystals. Later on, the group further gained insights into the influence of substituted position of DAT units on porosity. In detail, they prepared **H25a**–**25b** (see Figure 22e,f) bearing pentaerythrityltetraphenyl ethers as HOF backbones [20]. However, **H25a** (see Figure 23d for HOF geometry) grown from DMSO/dioxane possesses approximately 66% crystal space available for guests, whilst the counterpart **H25b** obtained from the same solvent exhibits only 57% porosity. We speculate that substitution position of DAT moieties has a significant impact on the association models within dimeric DAT-DAT species. In detail, DAT units in **H25a** tend to associate with neighboring motifs according to type A and B assembly models. In contrast, type B binding model is predominant in the analogous **H25b**. As an extension work, they further designed and prepared a versatile dendritic building motif **26** (see Figure 23a) which contains a dipentaerythritylhexaphenyl ether scaffold. Clearly, this elaborate design enables six DAT subunits to be compatible with six flexible arms in this scaffold [75]. Unexpectedly, crystalline **H26** (see Figure 23b) derived from such dendritic building blocks has larger micropore sizes and crystal space available for guests, determined to be up to an impressive 66%. Besides, X-ray diffraction investigations demonstrated that the supramolecular network (see Figure 23b) was held together via type A and C hydrogen bonding models. Although it is obvious that positioning molecules in controllable manners via hydrogen bonding is a great challenge at present, previous studies may provide us with empirical guidance to design HOFs:

(a) For neutral building motifs, the best position of a DAT motif generally lies in the phenyl *para*-position rather than *ortho-* and *meta-*positions, since a DAT motif in a *para*-position is more likely to enable scaffolds to separate from each other as far as possible when the building motifs are in close proximity to each other. As for charged building motifs, their complexation manners are rather difficult to predict precisely due to the subtle equilibrium between electrostatic and hydrogen bonding interactions.
(b) Even though high porosities may be achieved from the elaborate design of rigid building blocks, it is essential to “provide” DAT moieties or backbones with appropriate degree of flexibility for the purpose of rendering building motifs more adaptable to well-defined supramolecular networks;
(c) Despite the fact that enlarged building motifs are more likely to generate porous HOFs with better performance in terms of gas absorption or isolation [71], but it by no means indicates that HOFs with enlarged building motifs unambiguously result in remarkable enhancements in porosity. On the basis of these empirical principles, we can qualitatively elucidate the relationship between building motifs and porosities of following HOFs.

**Figure 24:**
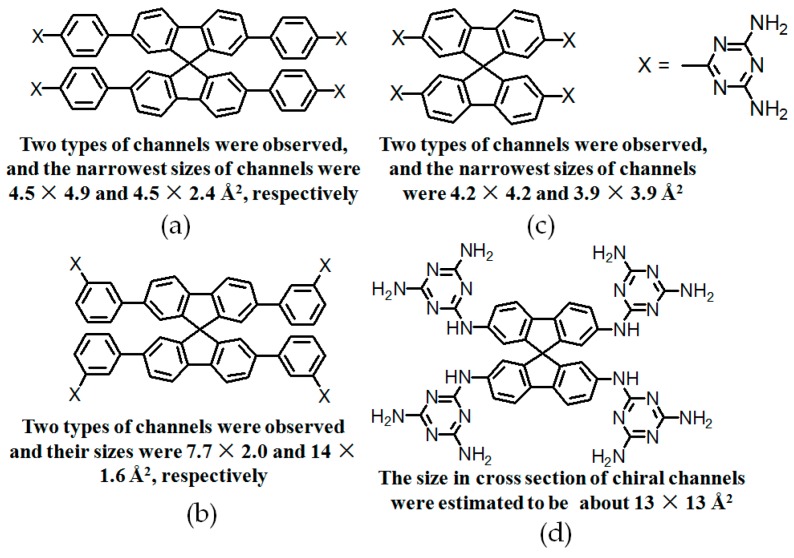
Structures of building motifs **27a** (**a**); **27b** (**b**); **27c** (**c**); and **27d** (**d**).

Although the synthesis of such a rigid backbone was a rather sophisticated task, building motifs bearing the 2,4,8,10-tetraoxaspiro[5,5]undecane backbone (see Figure 24a–d) were investigated as well [21]. When DAT units were linked to 2,2′,7,7′-tetrasubstituted spirobifluorene, approximately 60% of the pore volume of **H27c** was exploited to encapsulate solvent guests. However, integration of phenyl spacers into the spirobifluorene backbone did not improve the porosity. On the contrary, the pore volume accessible for guests further decreased to 53% and 44%, respectively, for both cases of **H27a** and **H27b**. Subsequently, the building motif **27d**, containing more flexible NH linkers between the sticky sides and backbone, was designed and synthesized. As manifested by theoretical simulation using the PLATON software, **H27d** possessed an impressive 75% crystal pore volume for guest inclusion, which is a record in the history of porous HOFs [76].

#### 2.7.2. Planar DAT-Based Building Blocks

**Figure 25:**
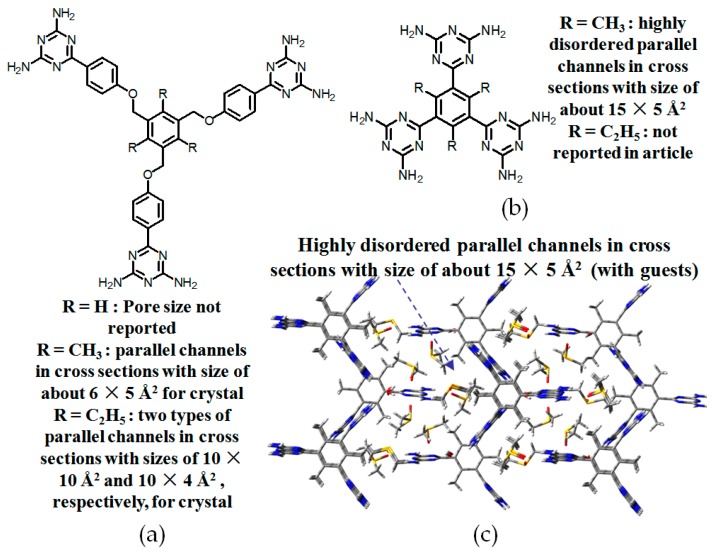
(**a**) Structures of motifs **28a** (R = H), **28b** (R = CH_3_), and **28c** (R = CH_3_CH_2_); (**b**) Structures of motif **29a** (R = CH_3_), and **29b** (R = CH_3_CH_2_); (**c**) Top view of **H29a** grown from DMSO/toluene.

**Figure 26:**
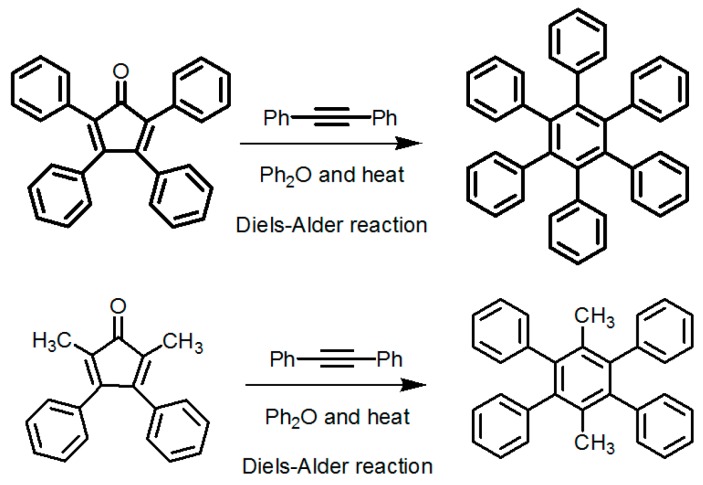
Synthesis routes of rigid cores in building motifs **30a** and **30b**.

**Figure 27:**
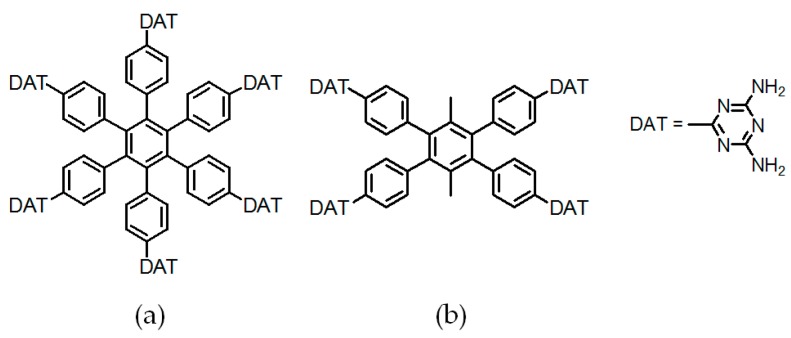
Structures of building motifs **30a** (Figure 27**a**), **30b** (Figure 27**b**).

**Figure 28:**
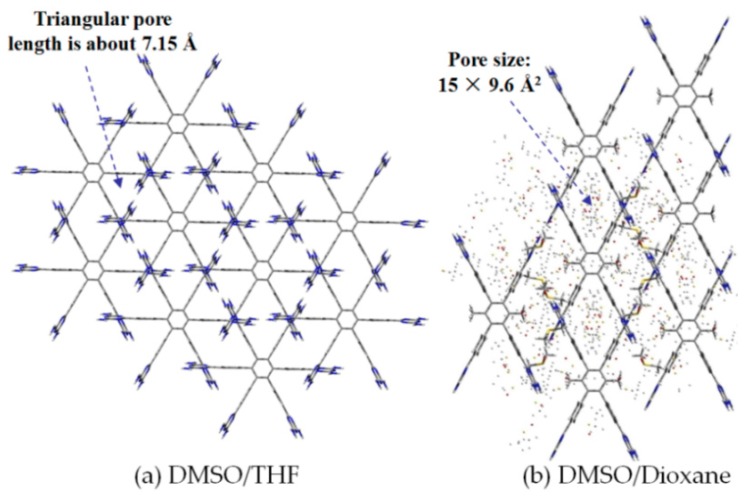
Top views of porous **H30a** (**a**) and **H30b** (**b**) grown from DMSO/THF and DMSO/dioxane.

By contrast, the self-complexation behaviors of planar DAT-based building motifs are relatively simple to predict and manipulate. Theoretically, motifs initially aggregate into planar or non-planar sheets by self-assembly of DAT moieties, whilst hydrogen bonding (type B or C association models, etc.) or other non-covalent interactions (π-π stacking or van der Waals force, etc.) are responsible for further extension of stacked supramolecular networks. It should be pointed out that other assembly models could not be excluded, but our attention is mainly paid to the most universal model for the purpose of elucidation convenience. In principle, detailed association models of HOFs are closely correlated with the conformational features of their building motifs and the surrounding organic media. For counterpart motifs **28a**–**28c** (see Figure 25a), the growth of crystalline HOFs was confirmed to obey the aforementioned association model. In detail, building motifs are held together by the potential conformations of dimeric DAT-DAT to define well-organized hydrogen-bonded sheets, while the assembly patterns of sheets are less predictable. Analogous to planar conjugated trimesic acid, all DAT groups in building motifs are almost cofacial with the central phenyl core although all sticky sides are linked with central cores via flexible CH_2_O moieties. As a consequence, **28a** merely take advantage of a type B assembly model to define planar hexameric rosettes, which further assemble into a less open network (32%) via interplanar π-π stacking of neighboring sheets, while in both cases of **28b**,**c**, it was found that the resulting hydrogen-bonded sheets preferred to select type A as the predominant conformation to accomplish self-assembly of two dimensional porous sheets, thereby self-complexation of sticky sides eventually lead to the formation of open hydrogen-bonded networks (44%–60%) [77]. Clearly, elimination of flexible CH_2_O linkers immediately give rise to more rigid building motifs **29a** and **29b** (see Figure 25b), which were also examined via supramolecular crystal engineering [78]. Nevertheless, a series of motifs obviously packed less efficiently as compared to **28a**–**28c**. For instance, XRD studies revealed that only **H29a** grown from DMSO/toluene media (see Figure 25c) can adopt both type A and C association patterns to form a hydrogen-bonded open network. In contrast, merely corrupted hydrogen bonding sheets were assembled together via interacting with encapsulated solvents when **H29a** and **H29b** were grown in DMSO/chlorobenzene and DMSO/toluene, respectively. Although the high polarity of DMSO severely interfered with the strength of hydrogen bonds, approximately 50%–60% of crystal volume was available for guests [78]. Wuest concluded that inefficient hydrogen bonding connections between these sheets may result from the compact design of the building motifs. As discussed above, HOFs assembled from *C*_3_-symmetrical DAT based building motifs show crystal porosities of no more than a mere 60%, which is still lower than the highest record of **H27d**. Further increasing the number of DAT sticky sides may seem an accessible approach to enhance the porosity of HOFs, as validated by systematic comparisons of the OH-containing HOFs in Table 1. In a related work, Wuest prepared two interesting enlarged building motifs **30a**,**b** (see Figure 27), which bear *C*_6_-symmetrical hexaphenylbenzene analogues and multiple DATs [79]. Accompanying the elimination of CO, all rigid cores were prepared via Diels-Alder cycloaddition reactions (see Figure 26) with decent yields. As anticipated, XRD investigations of **H30a** demonstrated that this building motif was capable of interacting with six neighboring motifs to define hydrogen bonded sheets with triangular micropores via a type A binding model. By virtue of type C binding models, hydrogen-bonded sheets of **H30a** are permitted to accomplish efficient crystal packing in mixtures of DMSO/THF, DMSO/toluene, or HCOOH/EtOH. In an exceptional case, stacking of sheets in DMSO/benzene is maintained by binding with encapsulated solvents, which is analogous to that of **H29b**. Additionally, the porosities of **H30a** crystals grown from DMSO/THF (see Figure 28a) and DMSO/benzene media were calculated to be impressive 70% and 72%, respectively, whereas **H30a** obtained from mixtures of DMSO/toluene and HCOOH/EtOH were relatively less opened (56%). It is obvious that the polarity of the solvents used has a significant impact on the crystal topologies.

Such a phenomenon is also confirmed by the crystalline networks of **H8** that are obtained from solvents with different components. However, it is still difficult to elucidate precisely how self-complexation of DAT moieties is affected by solvent polarity. Additionally, for porous **H30**, the outstanding porosity properties are not strongly correlated with the number of DAT sticky sides. In the case of motif **30b** (see Figure 27b), supramolecular assembly in DMSO/dioxane eventually gives rise to the formation of a highly ordered porous geometry (see Figure 28b for HOF geometry) with a highest porosity value of 75%.

**Figure 29:**
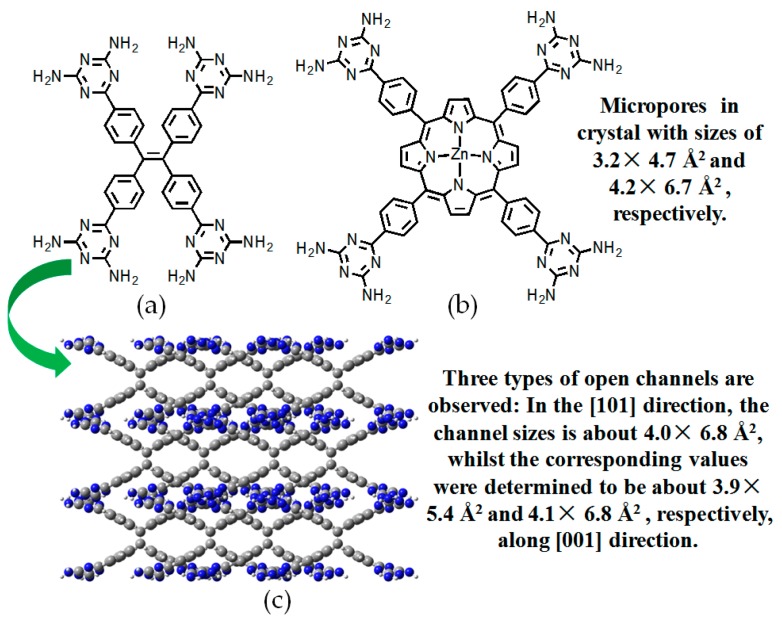
Structures of building motif **31** (**a**) and **32** (**b**); (**c**) Porous crystalline network of **H31**.

To expand application scope of planar rigid cores, tetrasubstituted ZnTDPP (zinc 5,10,15,20-tetrakistetra(4-cyanophenyl)porphyrin) and tetraphenylethylene (TPE) scaffolds were employed in order to explore porous materials with new functions [80,81]. It is well known that porphyrin and TPE derivatives have been extensively investigated in optoelectronic materials such as dye sensitized solar cells (DSSCs) [82] with high efficiency and aggregate induced emission (AIE) materials [83]. However, they were barely reported for the construction of HOF materials. Recently, Chen and his coworkers reported two representative counterparts, namely **H31** and **H32** (see Figure 29 for these building motifs) [80,81].

As expected, combinations of a type A self-assembly model and the unique structural feature of the building motif clearly facilitate the formation of hydrogen-bonded sheets with diamond micropores. Thereby, the two dimensional sheets were converted into close stacked architectures through distorted type C self-assembly of DAT units. XRD study of **H31** revealed that the HOF (see Figure 29c) displayed a 55.3% space volume accessible for encapsulation of guests. Both type A and C hydrogen bonding models were adopted in the intermolecular assembly to afford two dimensional porous networks. By virtue of non-covalent interactions such as π-π stacking and weak hydrogen bonds between neighboring networks, porous layers assemble in well-organized manners to afford crystalline **H32**. It is noteworthy that both **H31** and **H32** exhibited prominent advantages in gas sorption and separation in later studies. As described above, the DAT moiety contributes significantly to the further improvement of porous HOFs, and the porosity performance of HOFs could be tuned by attaching to well-organized rigid cores with diverse structural features. Nevertheless, other well-defined building blocks should also be investigated with the aim of gaining deeper insights into the relationships between porosities and the elaborate design of building motifs, thus providing theoretical guidance for the effective design of HOFs with potential applications.

#### 2.7.3. DAP Moieties as Hydrogen-Bonded Motifs for **HOFs**

**Figure 30:**
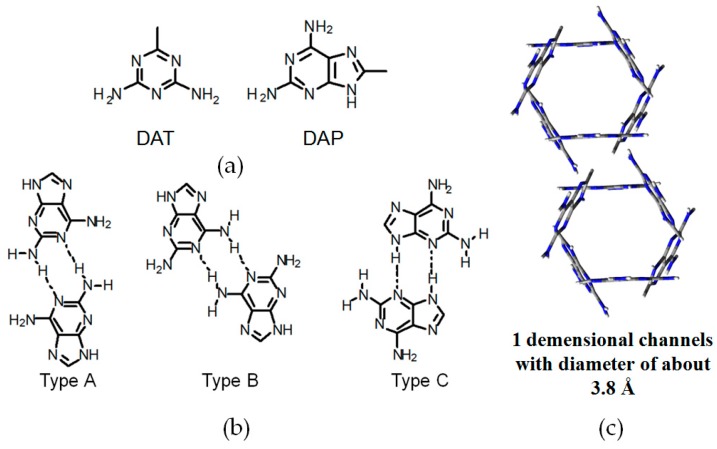
(**a**) Structures of DAT and DAP; (**b**) Potential hydrogen bonding patterns between DAP–DAP interactions; (**c**) Crystalline porous network of **H33c** grown from C_6_H_5_OCH_3_.

Evidently, the DAT unit has made significant contributions to the development of HOF materials over the past decade. Recently, Chen also reported another series of novel porous **H33a**–**33c**, which were constructed by utilizing DAP (2,6-diaminopurine) motif (see Figure 30a) [84]. Crystalline HOFs grown from various solvents (CH_3_OC_6_H_5_, DMF, and water) showed different self-complexation models, and the hydrogen-bonding assembly manners were determined by XRD analysis of the crystalline HOFs (see Figure 30c for HOF geometry). Considering that the structure and distribution of binding sites in the DAP motif is extremely similar to that of DAT, thereby it is reasonable that DAP exhibits highly similar complexation binding patterns (see Figure 30b) in solid or solution state.

#### 2.7.4. Potential Applications of DAT-Based HOFs

HOFs based on DAT moieties display unique advantages over MOFs in terms of gas storage or separation. Chen reported **H20**, the first example of an applicable HOF with a porous architecture, whose BET value was calculated to be 359.2 m^2^/g. In addition, it was found that **H20** has potential applications in C_2_H_2_ and C_2_H_4_ absorption [85]. As validated by absorption isotherm experiments, the separation selectivity between C_2_H_2_ and C_2_H_4_ at 273 K was determined to be approximately 7.6. This remarkable breakthrough motivated the research group to examine the enlarged tetrahedral motif **21**, which was presumed to assemble into porous HOFs with extended internal channels [71]. As expected, the resulting HOF indeed also exhibited excellent performance in the separation of C_2_H_4_/C_2_H_6_ and the selectivity value between C_2_H_4_/C_2_H_6_ even approach to incredible 14, which is superior to zeolite and artificial porous MOFs.

**Figure 31:**
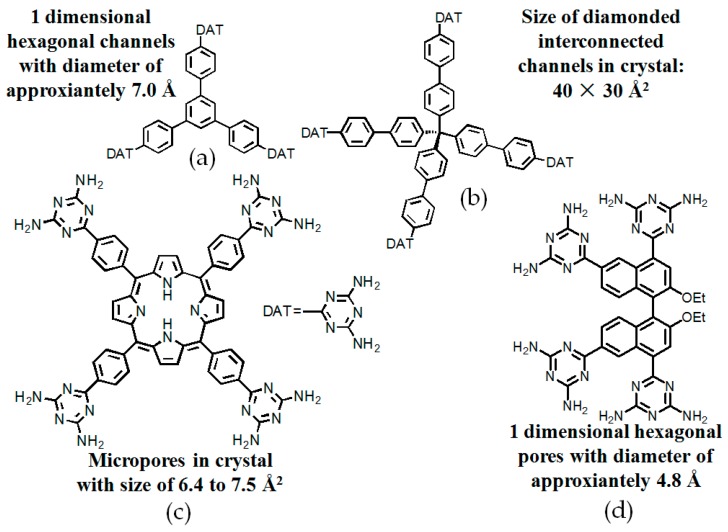
Structures of building motif **34** (**a**), **21** (**b**), **35** (**c**), and chiral **36** (**d**).

**Figure 32:**
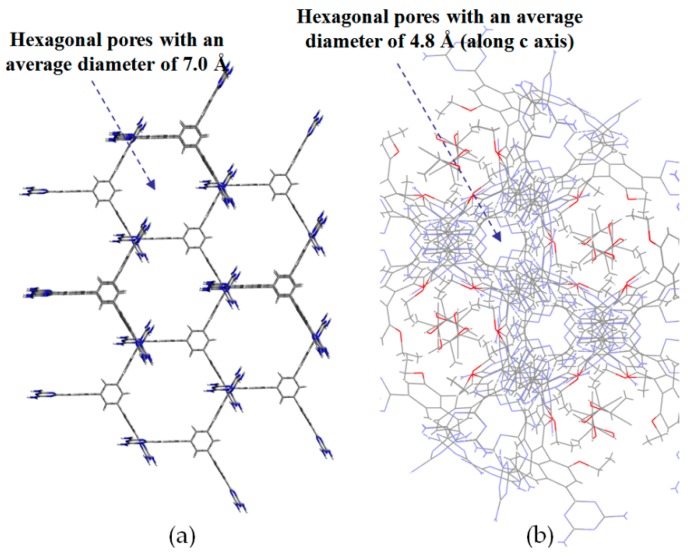
Crystalline Structures of HOFs assembled from building motif **34** (**a**) and chiral **36** (**b**).

The Chen group paid further attention to porous HOFs assembled from planar building motifs. Well-defined porous **H34** (see Figure 31a for building motif) displayed selectivity towards C_2_H_2_ and CO_2_ [86]. In order to gain deeper insights into its gas uptake behavior, they carried out theoretical calculations to investigate gas absorption. Crystalline activated **H34** was firstly optimized via a dispersion corrected DFT-D method and then absorption simulations were performed via Grand Canonical Monte Carlo (GCMC) software. As verified by simulation studies, the static binding energies of C_2_H_2_ and CO_2_ were estimated to be approximately 20.9 and 26.3 kJ/mol, respectively, indicating that this HOF exhibited stronger binding affinity toward C_2_H_2_. Inspired by the achievement of **H34** (see Figure 32a for crystalline geometry), various planar cores, including TPE, ZnTDPP, and H_2_TDPP (5,10,15,20-tetrakis(4-cyanophenyl)porphyrin) were linked to DAT moieties to give rise to **H31**, **H32**, and **H35** (see Figure 31c), respectively, with promising gas separation efficiencies [80,81,87]. For instance, benefitting from outstanding BET surface area (1101 m^2^/g) in its crystalline geometry, **H31** shows promising performance in CO_2_ or C_2_H_2_ gas absorption. In the case of C_2_H_2_, **H31** exhibited the highest absorption capacity record (around 101.7 cm^3^/g C_2_H_2_ gas under 296 K and 1 atm conditions). Analogously, this HOF also displayed high binding affinity toward CO_2_, and the measured CO_2_ uptake capacity was 90 cm^3^/g (296 K and 1 atm). Furthermore, **H31** was capable of acting as a functionalized molecular sensor in detecting aromatic compounds [88]. In contrast, absorption performances for **H32** and **H35** were less satisfactory despite the fact both HOFs exhibited remarkable declines in gas uptake. Probably due to the high similarity between structures of these building motifs (see Figure 29b and Figure 31c), not only were their BET values almost the same (124 m^2^/g for **H19** and 130.0 m^2^/g for **H35**), but also both HOFs displayed high selectivity toward CO_2_. **H35** also showed excellent proton conductivity performance (3.4 × 10^−6^ S cm^−1^), which was indeed rarely examined in the aforementioned HOF materials. Chen speculated that the amino protons in the DAT units may take part in the proton conduction [87].

Furthermore, molecular recognition has emerged as another critical potential application of porous HOF materials. Chen designed the first example of a DAT-based chiral HOF by covalently connecting DAT moieties to chiral (*R*)-BINOL to form chiral **H36** (see Figure 31d for the building motif) and successfully realized separation of racemic secondary alcohols for the first time [89]. Interestingly, it was found that no racemic primary alcohols could be efficiently isolated by **H36** (see Figure 32b for crystal geometry) due to the rather weak interactions between primary alcohols and the interior surfaces of **H36**. Accordingly, chiral secondary alcohols and aromatic alcohols were selected as guests for the purpose of gaining in-depth insight into the precise relationships between guest molecular size and enantioselectivity. As expected, **H36** demonstrated a promising *e.e.* value (92%, see Table 3) when it was utilized in the isolation of racemic 1-phenylethanol, while guests containing long aliphatic chains (C > 4) were relatively less favored (see Table 3). Besides, the BET value of **H36** was determined to be 237.6 m^2^/g, implying that **H36** may also act as an ideal candidate in gas storage or separation.

In addition, unique advantages of DAT has also attracted increasing attention in the formation of functionalized hydrogen bonding polymeric hydrogels aimed at medical applications such as drug delivery [90,91,92], or fluorescent probes for DNA bases [93,94,95]. Due to space limitation, these elegant examples are not included in this review.

### 2.8. Charge-Assisted Hydrogen Bonds for HOFs

Although well-defined DAT building blocks are capable of assembling into HOFs, sometimes neutral hydrogen bonds are not sufficiently robust to orient building motifs in predictable manners. To address this issue, some researchers have attempted to explore effective strategies to increase the strength of hydrogen bonding. In our perspective, the most attractive approach is to integrate charge-assisted hydrogen-bonding sites into building blocks. Charge-assisted hydrogen bonding, which aims at strengthening the noncovalent association of hydrogen bonds by introducing electrostatic interactions, provides a novel protocol to construct robust stimuli-responsive hydrogen bonding networks.

**Figure 33:**
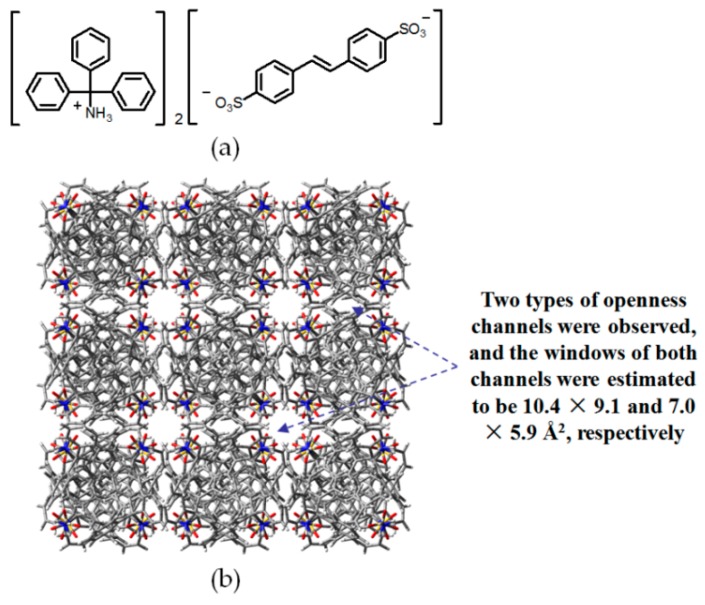
(**a**) Organic salt building motif of **H37**; (**b**) Crystalline porous network of **H37**.

**Figure 34:**
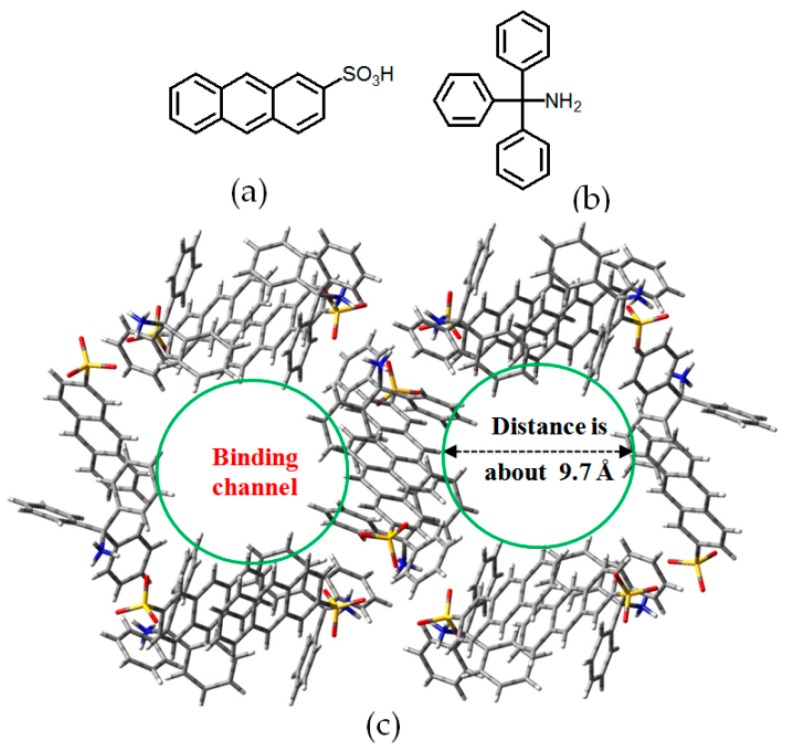
(**a**,**b**) Structures of building motifs 2-AS (left) and TPMA (right) in **H38**; (**c**) Crystalline porous network of **H38** (guests were omitted for clarity).

Tohnai and coworkers reported self-assembly of hydrogen-bonded porous **H37** by utilizing *trans*-stilbene-4,4′-disulfonic acid (SBDS) and triphenylmethanamine (TPMA) organic salts (see Figure 33a for structure of the organic salt) [96]. Interestingly, the HOF not only absorbed gas, but also showed nominal stimuli-responsive fluorescence upon intercalation of different guest molecules. The crystal structure of organic salt demonstrated that **H37** (see Figure 33b for crystalline geometry) possesses a maximum and minimum void space of 94.64 Å^2^ and 41.3 Å^2^, respectively, and the space volume is available for the inclusion of three *o*-chlorotoluene molecules at one time. More interestingly, the intercalated aromatic guests significantly affected the responsive fluorescence emission behaviors owing to their diverse ionization potentials. Additionally, isotherm experiments confirmed that the porous HOF selectivity absorbed CO_2_ with a capacity of 86.5 cm^3^/g (STP at *P*/*P*_0_ = 0.99). In addition, Tohnai reported a novel water-responsive **H38**, which was capable of releasing intercalated guests via adding water [97]. The porous HOF, which consists of cube-like subunits in crystalline geometry (see Figure 34c), is fabricated by multiple charge-assisted hydrogen bonding interactions between 2-sulfonaphthalene (2-AS) and TPMA (see Figure 34a,b for the structures of 2-AS and TPMA). In the crystalline cubes, the protons of 2-AS transfer to NH_2_ groups from organic salts via intermolecular charge-assisted N–H⋯O hydrogen bonds. Notably, various guests with appropriate size can be intercalated and released by adding water or exposing the guest-containing HOF under saturated water vapor conditions. More interestingly, the porous HOF displayed excellent performances in emission modulation, which might be regulated by controlling π-π overlapping extents between aromatic guests and aromatic moieties in building motifs. For instance, when 1,2,4-trichlorobenzene was encapsulated, the HOF material exhibited a red-shifted fluorescence emission band centered at 535 nm, but the intercalation of 1,3,5-trimethylbenzene immediately resulted in the blue-shifted emission band centered at 454 nm.

Recently, Wuest and co-workers also paid their attention to the charge-assisted hydrogen bonds and attempted to obtain HOFs. In their work, dicationic bis(amidine) analogues were deliberately selected as hydrogen bonding donors, which was able to complex with anionic species, including phosphonate, sulfonate, or carboxylate compounds. However, single crystals revealed that nearly all resulting crystalline geometries were indeed no porous even though hydrogen bonding interactions were significantly enhanced [98,99]. What’s more, it was found that the charge-assisted hydrogen bonding protocol can be exploited in the fabrication of functionalized supramolecular copolymers [100,101].

## 3. Conclusions

As discussed above, we have summarized eight categories of HOFs in this review. Indeed, the crucial roles of sticky sides cannot be ignored. Regardless of solvation effects, it is evident that DAT sticky side is more expert in architecting well-defined porous HOFs with higher porosities as compared to other types of hydrogen bonding groups. As shown in Table 1 and Table 4, due to the weak binding strength, the simplest hydrogen-bonded sticky sides (such as OH, NH_2_ or CONH_2_) are confirmed less efficient to induce the assembly of porous HOFs in predictable manners when they are covalently connected with **RB1**–**8**. Unambiguously, such sticky sides are too brittle to maintain polymeric topologies. It seems that an accessible approach to handle this issue is to integrate as many sticky sides as possible. However, such a design protocol is severely restricted by geometrical feature of scaffold. By contrast, the strong self-aggregated tendencies of either COOH or B(OH)_2_ group are capable of being utilized to further replace these weakened sticky sides. However, building motifs containing B(OH)_2_ groups were rarely investigated probably owing to inevitable dangerous and tedious preparation. To improve porosities, hydrogen-bonded dimers, including pyridone-pyridone, UPy-UPy, and DAT-DAT arrays, have been examined systematically. Among them, the DAT moiety is confirmed as the most promising sticky side for the assembly of HOF materials, since it cannot only be obtained by means of facile synthesis with decent yields, but is also compatible with a wide range of backbones via rational design protocols. More importantly, DAT units are capable of harnessing three potential dimeric conformations to facilitate self-complexation of DAT-containing building motifs. In contrast, integrations of pyridone subunits into HOF building motifs are rather tedious and less efficient. Although pyridone is also reliable to construct HOFs with high porosities, such HOFs generally from carboxylic acids evidently interfere with hydrogen-bonding association between sticky sides. UPy-UPy dimer exhibits the strongest self-association, but it seems that the highly flexible urea group poses a great threat to the formation of ordered HOF crystals. From our perspective, porosity properties do not rely heavily on the complexation abilities of hydrogen-bonded dimers. Other key factors, including solvent polarities, structural features, and the rigidity of building motifs, should also be seriously taken into consideration. Building motifs bearing tetrahedral spirobifluorene or planar hexaphenylbenzene motifs have stronger propensities to achieve desirable porosities and seem less sensitive to polarity changes of solvent media, as manifested by XRD studies of HOFs. However, it is still difficult to exemplify exactly how solvent polarities or solvation effects affect the formation of crystalline topologies at present. Particularly, HOFs from supramolecular macrocycles should be highlighted. Different from the residual sorts of building motifs, such motifs as pillar[n]arene or CB[6] show unique advantage in terms of selective gas absorption and storage. As manifested by crystalline geometries, macrocycle-containing HOFs can adopt either hydrogen-bonding directed stacking of macrocycles or combination of hydrogen bonding and exterior noncovalent interactions of macrocyclic surfaces to define open channels. Moreover, the intrinsic rigid electron-rich cavities provide more potential binding sites for intercalated guests with appropriate sizes. Accordingly, this category of HOFs generally has the potential to selectively capture specific small gas molecules, depending on the principles of the size complementary and the binding energies of gas molecules. Such principles are also applicable to other sorts of HOFs. As shown in Table 4, the category of dipepitide sequences indeed shows the lowest potentials in defining crystalline geometries with outstanding porosities. However, such building motifs have unique advantages in manipulating micropore and channel sizes by exploiting specific dipeptide sequences. Among all sticky sides, the charge-assisted hydrogen bonds clearly display the strongest strength. Interestingly, ionic natures of such hydrogen bonds endow resulting HOFs with unique response to water, which can be further employed to prepare stimuli-responsive porous HOF materials.

Further studies on crystalline HOFs will provide novel methodologies to direct the assembly of building motifs in controllable manners. Their applications in molecular recognition, gas storage or separation are also supposed to be further investigated. Additionally, theoretical computational software such as Gaussian, VASP, GCMC, PLATON, and Material Studio have provided the powerful tools to avoid irrational design of building motifs and valuable information about HOFs based on dispersion corrected semi-empirical PM7 or density functional theory (DFT) computations, including estimation of size and shape of micropores, or even predetermination of the most feasible crystalline structures of HOFs [102,103].

## Figures and Tables

**Table 1:** Porosities of HOFs.

Scaffold	Sticky Side = X	Porosity (Solvent System)
**RB1**	OH	No significant volume for guests (EtOAc)
**RB2**	OH NH_2_	Close packed (DMF/CHCl_3_) About 28% (CH_3_COOC_2_H_5_)
**RB3**	OH	No significant volume for guests (EtOAc)
**RB4**	NH_2_	<18% (DMF/water)
**RB5**	NH_2_	Close packed (benzene/CH_3_OH/hexane)
**RB6**	NH_2_	Close packed (THF)
**RB7**	NH_2_	Close packed (DMF/water)
**RB8**	CN	16% (EtOH), 40% (EtOAc), 57% (DMF), 58% (DMSO), 50% (1,4-dioxane), 50% (CH_3_CN)
**RB8**	OH [23]	48% (diethyl ether)
**RB8**	CONH_2_ [24]	48% (*n*-C_3_H_7_OH/water), 59% (DMSO), 7% (water)
**RB8**	COOH [25]	46% (MeOH)

**Table 2:** Average channel diameter and porosity in **H14a**–**14h**.

Entry	Average Channel Diameter	Porosity
AV (**H14a**)	5.36 Å	12.5%
VA (**H14b**)	5.08 Å	11.2%
AI (**H14c**)	4.3 Å	8.3%
VV (**H14d**)	4.0 Å	6.9%
IA (**H14e**)	3.6 Å	5.7%
IV (**H14f**)	3.4 Å	4.8%
VI (**H14g**)	3.0 Å	3.7%
LS (**H14h**)	4.3 Å	5.1%

**Table 3:** Enantiomeric excess (*e.e.*) of racemic secondary alcohols separated by **H36**.

Entry	Secondary Alcohols	*e.e.*Value
1	1-phenylethanol	92%
2	1-(4-chloropheny)ethanol	79%
3	2-butanol	77%
4	1-(3-chlorophenyl)ethanol	66%
5	2-pentanol	48%
6	2-hexanol	<10%
7	2-heptanol	<4%

**Table 4:** HOF porosities summarized in this review.

Category	HOF	Porosity
*2.1. Hydroxyl or amide groups as hydrogen-bonded motifs*	**H1** **H2** **H3** **H4** **H5**	>60% 52% 60% 64% not reported
*2.2. Carboxylic groups or pyrazole moieties as hydrogen-bonded motifs*	**H6** **H7**	38%–59% for **H6a**–**6d** 51%
*2.3. Amide or urea groups as hydrogen-bonded motifs*	**H8** **H9** **H10**	not reported 24% 13.7%
*2.4. Macrocyclic receptors as hydrogen-bonded motifs*	**H11** **H12** **H13**	not reproted 24.7% not reproted
*2.5. Linear dipeptide as hydrogen-bonded motifs*	**H14** **H15**	3.7%–12.5% not reproted
*2.6. Pyridone or UPy moieties as hydrogen-bonded motifs*	**H16** **H17** **H18** **H19**	60% 50% 24% not determined (powder)
*2.7. DAT or DAP moieties as hydrogen-bonded motifs*	**H20** **H21** **H22** **H23** **H24** **H25** **H26** **H27** **H28** **H29** **H30** **H31** **H32** **H33** **H34** **H35** **H36**	45% 42.5% 40%–50% 74% 60% for **H24a**, no crystals for **H24b**–**d** 66% (**H25a**) and 75% (**H25b**) 66% 53% (**H27a**), 44% (**H27b**), 60% (**H27c**), and 75% (**H27d**) 32% (**H28a**), 44% (**H28b**), and 60% (**H28c**) 56% (**H29a**) and 54% (**H29b**) ^[a]^ 72% (**H30a**) ^[a]^, 75% (**H30b**) ^[a]^ 55.3% (the value substantially decreased to 41.1% due to severely crystal contraction upon removal of guests) not reported not reported not reported 63.4% 54.3%
*2.8. Charge-assisted hydrogen-bonded motifs*	**H37** **H38**	31.5% not reported

^[a]^ The value refers to the highest porosity for HOFs assembled in diverse organic media.
